# The presence and impact of multimorbidity clusters on adverse outcomes across the spectrum of kidney function

**DOI:** 10.1186/s12916-022-02628-2

**Published:** 2022-11-01

**Authors:** Michael K. Sullivan, Juan-Jesus Carrero, Bhautesh Dinesh Jani, Craig Anderson, Alex McConnachie, Peter Hanlon, Dorothea Nitsch, David A. McAllister, Frances S. Mair, Patrick B. Mark, Alessandro Gasparini

**Affiliations:** 1 BHF Glasgow Cardiovascular Research Centre, Institute of Cardiovascular and Medical Sciences, University of Glasgow, 126 University Place, Glasgow, G12 8TA UK; 2 Department of Medical Epidemiology and Biostatistics, Karolinska Institutet, Stockholm, Sweden; 3 General Practice and Primary Care, Institute of Health and Wellbeing, University of Glasgow, Glasgow, UK; 4 School of Mathematics and Statistics, University of Glasgow, Glasgow, UK; 5 Robertson Centre for Biostatistics, Institute of Health and Wellbeing, University of Glasgow, Glasgow, UK; 6 Faculty of Epidemiology and Population Health, London School of Hygiene & Tropical Medicine, London, UK; 7 Public Health, Institute of Health and Wellbeing, University of Glasgow, Glasgow, UK

**Keywords:** Multimorbidity, Chronic conditions, Chronic kidney disease, Clustering analysis

## Abstract

**Background:**

Multimorbidity (the presence of two or more chronic conditions) is common amongst people with chronic kidney disease, but it is unclear which conditions cluster together and if this changes as kidney function declines. We explored which clusters of conditions are associated with different estimated glomerular filtration rates (eGFRs) and studied associations between these clusters and adverse outcomes.

**Methods:**

Two population-based cohort studies were used: the Stockholm Creatinine Measurements project (SCREAM, Sweden, 2006–2018) and the Secure Anonymised Information Linkage Databank (SAIL, Wales, 2006–2021). We studied participants in SCREAM (404,681 adults) and SAIL (533,362) whose eGFR declined lower than thresholds (90, 75, 60, 45, 30 and 15 mL/min/1.73m^2^). Clusters based on 27 chronic conditions were identified. We described the most common chronic condition(s) in each cluster and studied their association with adverse outcomes using Cox proportional hazards models (all-cause mortality (ACM) and major adverse cardiovascular events (MACE)).

**Results:**

Chronic conditions became more common and clustered differently across lower eGFR categories. At eGFR 90, 75, and 60 mL/min/1.73m^2^, most participants were in large clusters with no prominent conditions. At eGFR 15 and 30 mL/min/1.73m^2^, clusters involving cardiovascular conditions were larger and were at the highest risk of adverse outcomes. At eGFR 30 mL/min/1.73m^2^, in the heart failure, peripheral vascular disease and diabetes cluster in SCREAM, ACM hazard ratio (HR) is 2.66 (95% confidence interval (CI) 2.31–3.07) and MACE HR is 4.18 (CI 3.65–4.78); in the heart failure and atrial fibrillation cluster in SAIL, ACM HR is 2.23 (CI 2.04 to 2.44) and MACE HR is 3.43 (CI 3.22–3.64). Chronic pain and depression were common and associated with adverse outcomes when combined with physical conditions. At eGFR 30 mL/min/1.73m^2^, in the chronic pain, heart failure and myocardial infarction cluster in SCREAM, ACM HR is 2.00 (CI 1.62–2.46) and MACE HR is 4.09 (CI 3.39–4.93); in the depression, chronic pain and stroke cluster in SAIL, ACM HR is 1.38 (CI 1.18–1.61) and MACE HR is 1.58 (CI 1.42–1.76).

**Conclusions:**

Patterns of multimorbidity and corresponding risk of adverse outcomes varied with declining eGFR. While diabetes and cardiovascular disease are known high-risk conditions, chronic pain and depression emerged as important conditions and associated with adverse outcomes when combined with physical conditions.

**Supplementary Information:**

The online version contains supplementary material available at 10.1186/s12916-022-02628-2.

## Background

As the world’s populationlives longer, an increasing number of people are living with multiple chronic conditions (multimorbidity) [1]. These people suffer from high treatment burden as they often must cope with numerous medications and attend multiple specialists [2]. Multimorbidity is a leading challenge facing twenty-first-century medicine, and the optimal management of people with several complex medical conditions is yet to be established [3, 4].

Chronic kidney disease (CKD), defined as a persistent and irreversible degradation of kidney function, affects around 10% of the world’s population [5, 6]. Its multifactorial nature, progressive trajectory which is often associated with complications, and the development of cardiometabolic conditions mean that CKD is usually linked to multimorbidity. The care of people with CKD has been reported to be more complex than that of patients attending any other specialist [7] and they are disproportionately susceptible to adverse outcomes such as hospitalisation [8] and cardiovascular events [9]. Research into people with multiple chronic conditions has primarily focused on the number of conditions, and there has been less focus on clusters of conditions, particularly amongst people with CKD. Identifying clusters of conditions may help to improve the management of these people by informing preventative strategies and targeting treatments [10]. Some conditions may cluster together in clinically meaningful ways, such as if cluster membership tells us about common risk factors or if it helps stratify the risk of subsequent adverse events [11].

How multimorbidity changes with declining kidney function and how this contributes to adverse outcomes are not known. Clustering techniques can be used to uncover unknown patterns within data and are used in this study to identify clusters of conditions in two geographically distinct population-based cohorts. We identified these clusters in people at different levels of kidney function (including estimated glomerular filtration rate (eGFR) >60 mL/min/1.73m^2^) and studied the associated risk of mortality and major adverse cardiovascular events (MACE).

## Methods

### Study populations

We used two databases with anonymised health and administrative data: the Stockholm Creatinine Measurements project (SCREAM) covers the entire region of Stockholm, Sweden (approximately 2.9 million people during the study period) [12], and the Secure Anonymised Information Linkage Databank (SAIL) covers 79% of the population of Wales (approximately 3.4 million people during the study period) [13]. In both cohorts, primary care, secondary care, prescribing and mortality data were linked. We included adults with outpatient serum/plasma creatinine values after 1 January 2006. Calibrated laboratory analysers for creatinine were used in SCREAM; in SAIL, non-calibrated analysers may have been used and so creatinine values were multiplied by 0.95 to account for possible lack of calibration [14]. Participants were lost to follow-up if they permanently left the region for SCREAM or if they left a participating GP practice or the country for SAIL. Participants were followed up until 31 December 2018 in SCREAM and 1 June 2021 in SAIL.

### Selection of patients and kidney function thresholds

eGFR was calculated using the 2009 Chronic Kidney Disease Epidemiology Collaboration creatinine equation, but without considering the race coefficient [15]. We studied all adults with at least two eGFR values whose eGFR crossed one or more threshold during follow-up: 90, 75, 60, 45, 30 and 15 mL/min/1.73m^2^. All eGFR values were used to fit a linear mixed effects model, and this procedure is described in more detail in Additional file 1. By estimating the dates at which participants crossed these thresholds, we could define study covariates at these dates and outcomes thereafter. Participants could cross more than one eGFR threshold and could therefore be included in more than one eGFR category for subsequent analysis. Flow charts of included individuals are depicted in Additional file 1: Figs. S1A and S1B.

### Chronic conditions

For each participant, and at each eGFR threshold, we evaluated the presence of 27 different chronic conditions. In SCREAM, ICD-10 codes recorded in primary and secondary care records were used. In SAIL, ICD-10 codes were used for secondary care records with separate primary care read codes used, as previously described [8]. These conditions were ascertained using a validated algorithm [16] with some modifications: we excluded CKD as it was our exposure, and we used a single cancer definition (excluding non-melanoma skin cancer), combining lymphoma, metastatic cancer and non-metastatic cancer. Conditions were defined for each participant at the estimated date of crossing eGFR thresholds and time windows were applied as per the algorithm in use [16]. The cause of CKD was not incorporated as it is rarely possible to determine this from population-level data. As depression and chronic pain are poorly recorded in healthcare records, we enriched the definitions of these conditions with prescription data, as previously described [1, 8]. In brief, a participant was assigned to have depression if they had four or more antidepressant prescriptions within a year and chronic pain if they had four or more prescriptions for painkillers within a year (including antiepileptic medications such as gabapentin, so long as the participant did not have epilepsy).

### Outcomes

After identifying clusters of conditions, we studied associations between cluster membership and subsequent adverse outcomes. Outcomes were identified from death and secondary care records: all-cause mortality, MACE (myocardial infarction, stroke or cardiovascular death, denoted as MACE3) and MACE plus heart failure hospitalisation (denoted as MACE4). Relevant ICD-10 codes are available in Additional file 1: Table S1. To capture as many events as possible in both cohorts, the secondary care records used were from hospitals in Sweden and Wales that provide universal coverage.

### Statistical analysis

Baseline characteristics including the prevalence of chronic conditions were compared between participants in each eGFR category. Categorical variables were expressed as frequencies with percentages and continuous variables as medians with interquartile intervals (IQI). We compared the participants with available eGFRs who were included in the analysis to those with available eGFRs not included in terms of their birth dates, sex and number of eGFR measurements.

We applied a k-modes algorithm within each eGFR category to identify clusters of conditions [17]. This clustering technique identifies clusters of participants with similar combinations of covariates, in our case the 27 chronic conditions, maximising homogeneity within clusters and heterogeneity between clusters. We chose to use this algorithm as it can perform clustering with categorical data and is computationally efficient (given our large sample sizes). We ran the algorithm for two to 10 possible clusters, as we deemed a larger number of clusters not clinically useful. We allowed for a maximum of 20 iterations of the algorithm. The optimal number of clusters was selected using the elbow method, to minimise the within-cluster distance while selecting a parsimonious number of clusters [18]. We plotted gradients of the elbow plots to ease the choice, as gradients approach zero when the elbow plots flatten. The k-modes algorithm was repeated with participants stratified by age (< and ≥65 years).

The prevalence of chronic conditions in each cluster was compared to their prevalence in the overall eGFR category. Observed/expected (O/E) ratios were calculated by dividing condition prevalence in a cluster by the prevalence in each eGFR category. Prominent conditions for each cluster were identified as conditions which were common (≥20% prevalence) and more common than the overall eGFR category (O/E ratio ≥2) [19]. To prevent cluster descriptions becoming protracted, a maximum of three prominent conditions were selected as the defining condition(s) for each cluster, with the most prevalent conditions used if more than three were identified. To help compare the prominent conditions, the clusters were further categorised using the single most prevalent condition in each cluster, using that condition’s body system: cancer, cardiovascular, dermatological, endocrine, gastrointestinal, mental health and pain, neurological, respiratory, rheumatological and non-specific. We then compared the proportion of participants in clusters in each eGFR category.

Cluster allocation for all participants was fully determined prior to analysing the outcome data. We calculated crude rates of incident adverse events per cluster and expressed them per 1000 person-years at risk. Then, relationships between cluster membership and outcomes were assessed using Cox proportional hazard models, adjusting for age and sex. For the MACE analyses, participants were censored on the date of death. The reference groups were participants in clusters with no prominent conditions (based on prevalence). If there was more than one cluster with no prominent condition, the cluster with the highest number of participants was selected as the reference group. For each model, we tested the statistical significance of the clustering variable using Wald tests and produced standardised survival curves (using regression standardisation [20]) to quantify absolute risks for each cluster at each eGFR level considered in the study. We assessed the prediction of outcomes via internal validation of our models using time-varying area under the receiver operating characteristic curve (AUC) and Brier scores over the duration of follow-up; non-parametric bootstrap with 100 resamples was used to calculate standard errors for each metric. Models with age and sex only were compared to models which added cluster membership and models which added the number of chronic conditions.

Statistical analyses were conducted using R version 4.0.5 or later [21] with the tidyverse, nephro, lme4, SCREAM, klaR, glue, formattable, survival, broom, aod, ggalluvial, matrixStats, ggrepel, stdReg, ggtext, hrbrthemes, knitr, patchwork, readxl, riskRegression and cowplot packages. Code is available on Github for others to replicate our analysis: https://github.com/ellessenne/multimorbidity-ckd-clustering.

## Results

### Baseline characteristics

The SCREAM cohort consisted of 404,681 unique participants (53.5% women). The median age was lowest in the eGFR 90 category (58.8 years, IQI: 49.3–66.2) and highest in the eGFR 30 category (82.5 years, IQI: 73.7–88.4) (Table 1). The SAIL cohort consisted of 533,362 unique participants (55.3% women). The median age was lowest in the eGFR 90 category (55.5 years, IQI: 46.6–63.5) and highest in the eGFR 30 category (80.8 years, IQI: 73.1–86.5) (Table 2). Comparing both tables shows that SAIL participants in this study have a higher multimorbidity count when compared to their Swedish counterparts.

**Table 1:** SCREAM baseline characteristics by eGFR category

		Estimated glomerular filtration rate (eGFR) category (mL/min/1.73m^2^)					
		90	75	60	45	30	15
Number of participants		211,046	154,327	79,413	37,163	12,821	2953
Age (years)	Median (IQI)	58.8 (49.3 to 66.2)	68.7 (60.9 to 75.8)	76.7 (69.8 to 83.1)	81.4 (74.2 to 87.1)	82.5 (73.7 to 88.4)	74.7 (64.0 to 83.9)
Sex	Female (%)	107,965 (51.16)	83,321 (53.99)	44,061 (55.48)	20,497 (55.15)	6500 (50.70)	1172 (39.69)
Chronic condition count (% for eGFR category)	0	88,122 (41.8)	43,897 (28.4)	11,684 (14.7)	2858 (7.7)	591 (4.6)	70 (2.4)
	1	63,136 (29.9)	44,508 (28.8)	18,334 (23.1)	5903 (15.9)	1361 (10.6)	231 (7.8)
	2	34,152 (16.2)	31,654 (20.5)	18,645 (23.5)	7852 (21.1)	2048 (16.0)	461 (15.6)
	3	15,289 (7.2)	18,202 (11.8)	13,784 (17.4)	7493 (20.2)	2387 (18.6)	523 (17.7)
	4+	10,347 (4.9)	16,066 (10.4)	16,966 (21.4)	13,057 (35.1)	6434 (50.2)	1668 (56.5)

**Table 2:** SAIL baseline characteristics by eGFR category

		Estimated glomerular filtration rate (eGFR) category (mL/min/1.73m^2^)					
		90	75	60	45	30	15
Number of participants		214,798	204,053	129,174	68,036	24,412	4334
Age (years)	Median (IQI)	55.5 (46.6 to 63.5)	66.6 (58.5 to 73.8)	74.2 (67.5 to 80.5)	79.0 (72.4 to 84.6)	80.8 (73.1 to 86.5)	76.0 (65.9 to 83.8)
Sex	Female (%)	117,993 (54.9)	110,615 (54.2)	70,649 (54.7)	37,082 (54.5)	12,702 (52.0)	1870 (43.1)
Chronic condition count (% for eGFR category)	0	45,135 (21.0)	27,180 (13.3)	8085 (6.3)	1721 (2.5)	379 (1.6)	60 (1.4)
	1	61,264 (28.5)	47,732 (23.4)	21,402 (16.6)	7472 (11.0)	1895 (7.8)	307 (7.1)
	2	46,572 (21.7)	45,869 (22.5)	27,560 (21.3)	12,447 (18.3)	3578 (14.7)	624 (14.4)
	3	28,807 (13.4)	34,165 (16.7)	25,475 (19.7)	13,528 (19.9)	4579 (18.8)	824 (19.0)
	4+	33,020 (15.4)	49,107 (24.1)	46,652 (36.1)	32,868 (48.3)	13,981 (57.3)	2519 (58.1)

**Fig. 1:**
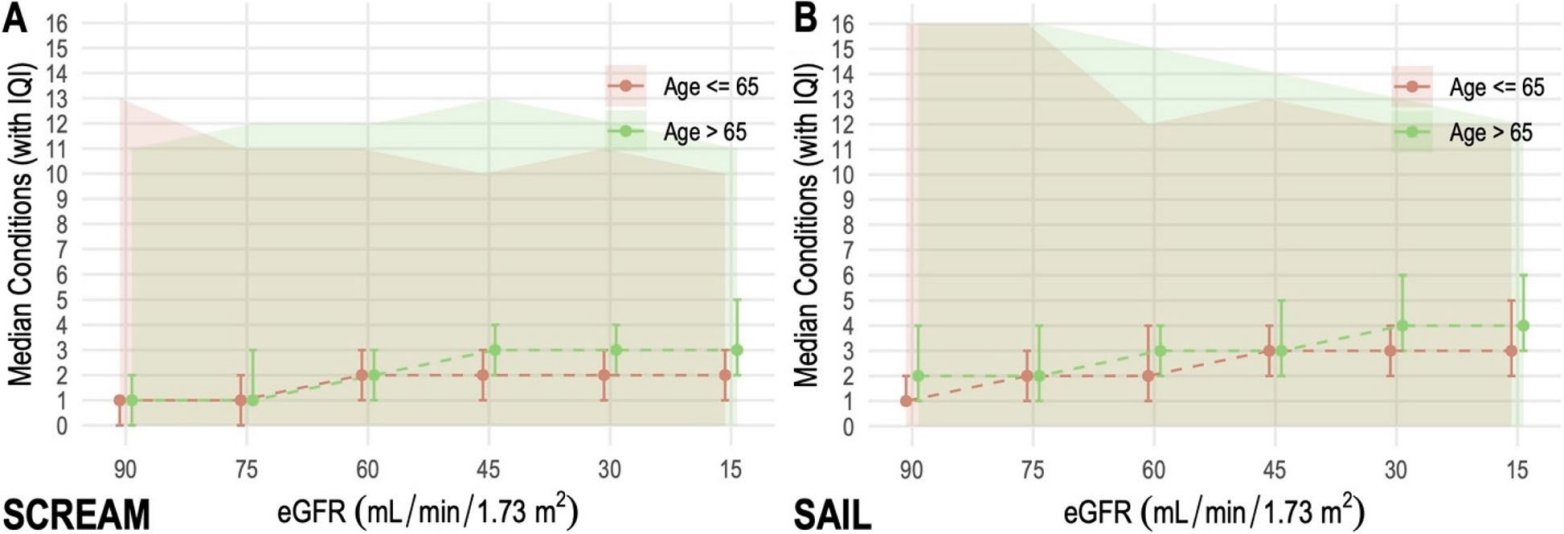
Median number of chronic conditions by cohort and eGFR category: **A** SCREAM and **B** SAIL. Error bars represent IQIs and the shaded areas minimum and maximum counts

Participants in the low eGFR categories had the highest number of chronic conditions, particularly in those aged over 65 years (Fig. 1). Participants included in the analysis tended to be born at earlier dates and have more eGFR measurements than those excluded (Additional file 1: Figs. S2A and S2B). The proportions of females and males included were similar, except at eGFR 15mL/min/1.73m^2^, where proportionally fewer women were included.

### Prevalence of chronic conditions by eGFR

The prevalence of most chronic conditions increased in lower eGFR categories (Additional file 1: Fig. S3). For example, in SAIL, the prevalence of cancer at eGFR 90 was 9.3% and at eGFR 15 25.7%.

The most frequently recorded chronic condition in SCREAM was hypertension, which ranged from 20.6% in the eGFR 90 group to 78.1% in the eGFR 15 group. Analogously, chronic pain ranged from 29.7% in the eGFR 90 group to 43.2% in the eGFR 15 group; diabetes ranged from 8.4 to 37.8% for eGFR 90 and 15; and heart failure ranged from 1.6 to 27.8% for eGFR 90 and 15.

The most frequently recorded chronic condition in SAIL was also hypertension, which ranged from 34.4 to 86.1% for eGFR 90 and 15, respectively. Analogously, chronic pain ranged from 21.5% for eGFR 90 to 38.4% for eGFR 15; diabetes ranged from 17.5 to 53.4% for eGFR 90 and 15. The proportion of participants with depression ranged from 35.0% for eGFR 90 to 28.7% for eGFR 15.

The optimal number of clusters varied at each eGFR level. Elbow plots (Additional file 1: Fig. S4) and gradient plots (Additional file 1: Fig. S5) suggested that the model fitness stabilised in each eGFR level at between five and nine clusters, i.e. using more clusters did not significantly improve the goodness of fit. Overall, the optimal number of clusters was highest at eGFRs 15 and 30 mL/min/1.73m^2^.

### Prevalence of conditions by cluster

**Fig. 2:**
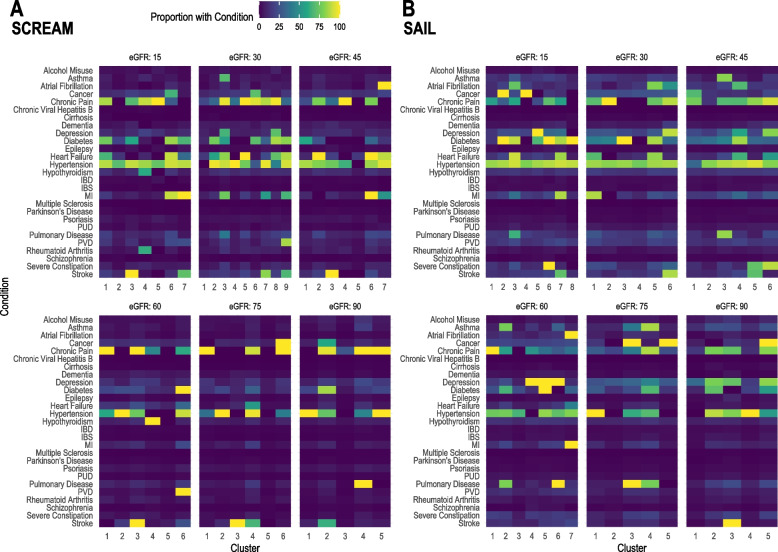
Heatmaps of chronic condition prevalence by cluster and eGFR category. **A** SCREAM. **B** SAIL. IBD inflammatory bowel disease, IBS irritable bowel syndrome, MI myocardial infarction, PUD peptic ulcer disease, PVD peripheral vascular disease

Table S2 in Additional file 1 shows the prevalence of chronic conditions in each cluster, simplified graphically in heatmaps (Fig. 2). Hypertension, diabetes and chronic pain were common in many clusters.

### Prominent conditions (based on prevalence)

**Fig. 3:**
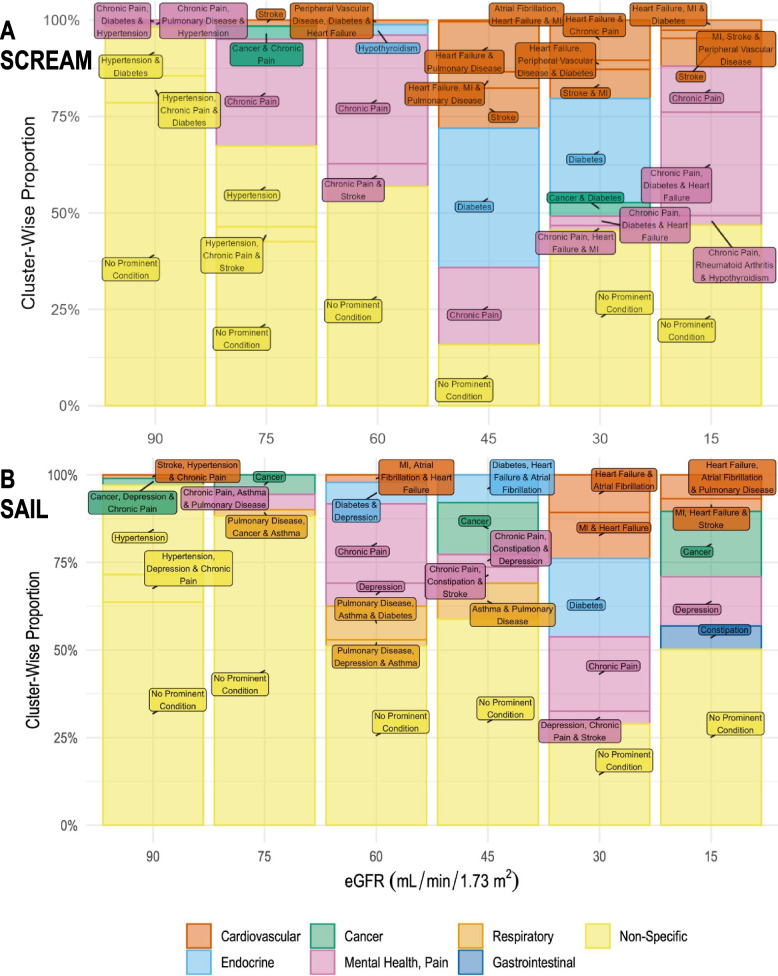
Proportion of patients in clusters by prominent conditions and body system. **A** SCREAM. **B** SAIL

Figure S6 in Additional file 1 depicts how prominent conditions were identified within each cluster. Although hypertension was the commonest condition in each eGFR category, it could not be a prominent condition in most of the clusters because the background prevalence was >50% and the O/E ratio therefore could not be ≥2. Tables 3 and 4 summarise the number of participants and the prominent condition(s) in each cluster. Some clusters in the same eGFR category share the same description, but are distinct because there are differences between the conditions separate to the “prominent” conditions. For example, at eGFR 15 in SAIL, there were two “Cancer” clusters, but in one cluster all participants had diabetes and in the other no participants had diabetes. Figure 3 shows the proportion of participants in each cluster by eGFR category. In both cohorts, most participants were included in one or two clusters with no system-specific prominent conditions, i.e. there was either no prominent condition or hypertension was the most prominent condition. The cluster-wise proportion of participants with no prominent condition, however, decreased as kidney function declined. As expected, diabetes and cardiovascular conditions featured more prominently as eGFR worsened. Chronic pain and, in SAIL, depression featured in clusters across the spectrum of kidney function.

**Table 3:** Prominent conditions by cluster in SCREAM

**SCREAM**				
**eGFR category**	**Cluster number**	***n***	**% of eGFR category**	**Cluster (defined via prominent condition(s))**
**15**	2	1383	46.8	No prominent condition
	1	790	26.8	Chronic pain, diabetes and heart failure
	5	354	12.0	Chronic pain
	3	212	7.2	Stroke
	6	77	2.6	Heart failure, MI and diabetes
	4	74	2.5	Chronic pain, rheumatoid arthritis and hypothyroidism
	7	63	2.1	MI, stroke and peripheral vascular disease
**30**	2	3455	26.9	Diabetes
	4	3259	25.4	No prominent condition
	1	2582	20.1	No prominent condition
	5	1338	10.4	Heart failure and chronic pain
	7	968	7.6	Stroke and MI
	6	453	3.5	Cancer and diabetes
	8	314	2.4	Chronic pain, diabetes and heart failure
	9	303	2.4	Heart failure, peripheral vascular disease and diabetes
	3	150	1.2	Chronic pain, heart failure and MI
**45**	1	13,421	36.1	Diabetes
	4	7426	20.0	Chronic pain
	5	5897	15.9	No prominent condition
	2	4837	13.0	Heart failure and pulmonary disease
	3	3855	10.4	Stroke
	6	1576	4.2	Heart failure, MI and pulmonary disease
	7	152	0.4	Atrial fibrillation, heart failure and MI
**60**	1	26,463	33.3	Chronic pain
	2	22,992	29.0	No prominent condition
	5	22,182	27.9	No prominent condition
	3	4667	5.9	Chronic pain and stroke
	4	2151	2.7	Hypothyroidism
	6	959	1.2	Peripheral vascular disease, diabetes and heart failure
**75**	5	65,668	42.6	No prominent condition
	1	42,753	27.7	Chronic pain
	2	32,312	20.9	Hypertension
	4	5961	3.9	Hypertension, chronic pain and stroke
	6	5187	3.4	Cancer and chronic pain
	3	2447	1.6	Stroke
**90**	3	165,836	78.6	No prominent condition
	1	26,586	12.6	Hypertension and diabetes
	5	14,681	7.0	Hypertension, chronic pain and diabetes
	4	2503	1.2	Chronic pain, pulmonary disease and hypertension
	2	1440	0.7	Chronic pain, diabetes and hypertension

**Table 4:** Prominent conditions by cluster in SAIL

**SAIL**				
**eGFR category**	**Cluster number**	***n***	**% of eGFR category**	**Cluster (defined via prominent condition(s))**
**15**	1	1656	38.2	No prominent condition
	5	608	14.0	Depression
	8	519	12.0	No prominent condition
	2	452	10.4	Cancer
	4	357	8.2	Cancer
	3	294	6.8	Heart failure, atrial fibrillation and pulmonary disease
	6	289	6.7	Constipation
	7	159	3.7	MI, heart failure and stroke
**30**	4	7065	28.9	No prominent condition
	3	5452	22.3	Diabetes
	2	5197	21.3	Chronic pain
	1	3212	13.2	MI and heart failure
	5	2614	10.7	Heart failure and atrial fibrillation
	6	872	3.6	Depression, chronic pain and stroke
**45**	2	40,000	58.8	No prominent condition
	1	10,056	14.8	Cancer
	3	6990	10.3	Asthma and pulmonary disease
	4	5391	7.9	Diabetes, heart failure and atrial fibrillation
	5	3123	4.6	Chronic pain, constipation and stroke
	6	2476	3.6	Chronic pain, constipation and depression
**60**	3	66,079	51.2	No prominent condition
	1	29,274	22.7	Chronic pain
	2	12,383	9.6	Pulmonary disease, asthma and diabetes
	4	8486	6.6	Depression
	5	7918	6.1	Diabetes and depression
	6	2310	1.8	Pulmonary disease, depression and asthma
	7	2724	2.1	MI, atrial fibrillation and heart failure
**75**	1	99,957	47.0	No prominent condition
	2	84,041	41.2	No prominent condition
	5	11,278	5.5	Cancer
	4	9023	4.4	Chronic pain, asthma and pulmonary disease
	3	3754	1.8	Pulmonary disease, cancer and asthma
**90**	1	136,801	63.7	No prominent condition
	4	54,820	25.5	Hypertension
	2	16,909	7.9	Hypertension, depression and chronic pain
	5	4119	1.9	Cancer, depression and chronic pain
	3	2149	1.0	Stroke, hypertension and chronic pain

When clustering was stratified by age, proportionally more participants aged over 65 years were in clusters with prominent conditions (based on prevalence) compared to those under 65 years (Additional file 1: Figs. S7A and S7B). Clusters which featured heart failure and myocardial infarction existed at eGFRs 30 and 45 in both cohorts and in all age groups, but these were proportionally larger in those over the age of 65 than under 65. In SAIL, cancer featured in more clusters in those over the age of 65 than under 65.

### Outcomes

In SCREAM, the median follow-up time ranged from 1.94 years (IQI 1.87–2.03) at eGFR 15 to 6.32 years (IQI 6.30–6.34) at eGFR 90 (Additional file 1: Table S3A). In SAIL, the median follow-up time ranged from 5.51 years (IQI 5.32–5.68) at eGFR 15 to 7.45 years (IQI 7.42–7.47) at eGFR 90 (Additional file 1: Table S3B). In both cohorts, crude event rates were higher at lower eGFR categories compared to higher eGFR (Additional file 1: Fig. S8). Event rates were lowest in clusters with no prominent condition.

Clustering membership was significantly associated with event rates for each outcome (Wald test *p*-values < 0.001 in all eGFR categories, adjusted for age and sex). This was reflected in the standardised survival curves at every eGFR level (Additional file 1: Fig. S9). Finally, the predictive performance (of predicting adverse outcomes) of cluster membership information was, overall, similar to that of using the number of conditions (AUCs displayed in Additional file 1: Fig. S10 and Brier scores in Additional file 1: Fig. S11).

**Fig. 4:**
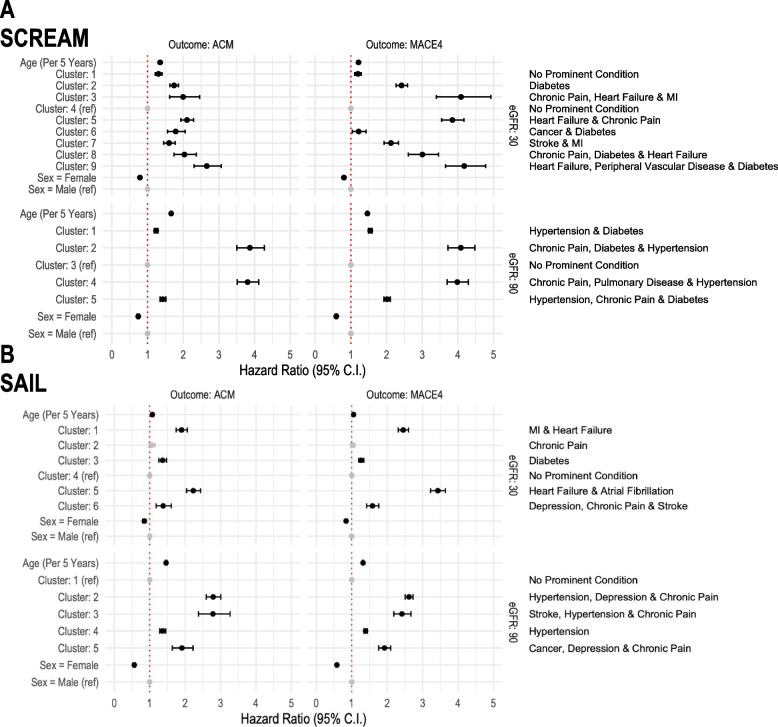
Forest plot showing the risk of all-cause mortality (ACM) and MACE by cluster allocation. **A** SCREAM. **B** SAIL. Hazard ratios are adjusted for sex and age, and on the right side, the prominent condition of each cluster is listed

The relative rates of all-cause mortality and MACE were highest in the clusters with cardiometabolic prominent conditions (Additional file 1: Fig. S12, Additional file 1: Table S4). Figure 4 features results from low (30) and high (90) eGFR categories. In SAIL at eGFR 30, cluster 5 (heart failure and atrial fibrillation) showed a hazard ratio (HR) for all-cause mortality of 2.23 (95% confidence interval (CI) 2.04–2.44) and for MACE HR 3.43 (CI 3.22–3.64).

Hazard ratios tended to be higher when cardiometabolic conditions were combined with chronic pain or depression. In SCREAM at eGFR 90, cluster 1 (hypertension and diabetes) showed an HR for all-cause mortality of 1.24 (CI 1.19–1.29) and for MACE HR 1.54 (CI 1.49–1.60). Also, at eGFR 90 in SCREAM, cluster 2 (in which chronic pain was prominent in *addition* to hypertension and diabetes), the HR for all-cause mortality was 3.87 (CI 3.51–4.27) and MACE 4.08 (CI 3.72–4.48).

However, when chronic pain or depression was the sole prominent condition, these clusters were either not at increased risk of adverse outcomes or the increased risk was minimal. For example, in SCREAM at eGFR 60, cluster 1 (chronic pain) all-cause mortality HR was 1.11 (CI 1.07–1.14) and MACE HR 1.14 (CI 1.11–1.17). In SAIL at eGFR 60, cluster 4 (depression) all-cause mortality HR was 0.97 (CI 0.89–1.05) and MACE HR 1.02 (CI 0.97–1.07).

## Discussion

In two geographically distinct health systems, we report the following findings: (1) low eGFR is accompanied by increasing age and increasing prevalence of chronic conditions; (2) these chronic conditions often cluster, with differential patterns across the eGFR spectrum, and show strong associations with the risk of adverse outcomes; (3) clusters with cardiovascular conditions were more prominent at low eGFR; (4) chronic pain and depression were common and, when combined with physical conditions, were associated with adverse outcomes; (5) clustering information could predict the risk of adverse outcomes in a similar way to the number of chronic conditions, with the advantage of being more clinically relevant. Collectively, these findings illustrate the complexity of medical conditions for people with CKD and have practical implications for service delivery, by supporting a move away from healthcare for individual diseases towards the development of clinical guidelines for common clusters of conditions.

In both cohorts, there was a dichotomy between low-risk clusters with low rates of chronic conditions and high-risk clusters featuring cardiovascular conditions. This agrees with an analysis of people with CKD in the Chronic Renal Insufficiency Cohort Study which found one large cluster with relatively healthy individuals [22] and a longitudinal study which found that as clusters were compared over follow-up, cardiovascular conditions became prominent as the participants aged [23]. Our finding that cardiovascular clusters became more dominant at eGFR 30 and 15 mL/min/1.73m^2 ^was not surprising, as people with CKD, diabetes and heart disease are a well-recognised group with consistently poor outcomes[24]. This group of patients may benefit from integrated clinics, where multiple specialties see patients together. For example, clinics with cardiology, nephrology and endocrinology have been found to be effective at optimising treatment (e.g. improving glycated haemoglobin levels and commencing sodium-glucose co-transporter-2 inhibitors) [25]. There is evidence that integrated clinics may help address some of the problems associated with attending hospital clinics, such as by reducing the number of appointments patients must attend and by improved continuity of care [26]. However, the impact on quality of life is less clear [27] and further work is required to determine if these models of care work well. As these clusters in our study were at heightened risk of adverse events, they may benefit from targeted evidence-based interventions such as statins [28], renin-angiotensin system inhibitors [29–32], sodium-glucose co-transporter-2-inhibitors [33, 34] and smoking cessation support [35].

Chronic pain was common in both cohorts, particularly at low eGFR, and identified in many clusters. This agrees with a recent systematic review reporting that chronic pain was common in people with CKD[36]. The systematic review reported a higher prevalence of chronic pain (48%) [36] compared to our cohorts, perhaps because estimates were based on clinical studies with assessment of pain scales, and our estimates may be affected by poor recognition of pain by health professionals. We in part used prescribing data to identify chronic pain and depression, and a reluctance amongst clinicians to prescribe nephrotoxic medication may have led some patients with chronic pain to go undetected. It also agrees with a clustering analysis of people with multimorbidity in England that found chronic pain to feature in 13 of 20 clusters and to be associated with frequent health service use [37]. We similarly found that adverse outcome rates were higher when chronic pain featured in clusters alongside physical conditions, but not on its own. Management of chronic pain is challenging, especially in people with CKD. Prescribers often avoid non-steroidal anti-inflammatory drugs because of nephrotoxic effects, and both these medications and opioids are associated with significant harm [38]. Previous studies of multimorbidity in people with CKD have not explored the importance of chronic pain [39, 40]. More research must therefore be done to understand why its prevalence in CKD is so high and what can be done to improve its management.

Depression featured in clusters in SAIL, often alongside physical conditions. Mental and physical conditions are known to occur together frequently, but treatment in these people can be challenging. Clusters in our study which featured depression in combination with physical conditions were associated with an increased risk of adverse outcomes, which is consistent with previous studies [41]. Depression in people with CKD is currently under-recognised and under-treated, and antidepressant medications do not work as well as when kidney function is normal [42]. In a systematic review of interventions for people with multimorbidity, those targeting depression were the most effective, particularly alterations to care delivery, such as nurses and psychologists setting goals with patients [43]. Interventions like these therefore warrant investigation in people with CKD and multimorbidity.

We found that clustering conditions did not significantly improve the prediction of outcomes over counting conditions. This is consistent with a study of over 8 million English people, which could not identify any clusters which could be targeted to reduce emergency hospitalisations [44]. However, our study was not aimed at developing a prediction model for the risk of adverse outcomes and metrics were only internally validated, thus limiting our conclusions with regard to the predictive ability of clusters versus condition counts. Rather than being incorporated into risk stratification, clusters of conditions may be more helpful in informing preventative measures and clinical guidelines. For example, public health measures might encourage healthy lifestyles to reduce the numbers of people in high-risk clusters, e.g. those with CKD, diabetes and heart disease. Clinical guidelines could be developed to help clinicians treat chronic pain amongst people with CKD and cardiometabolic conditions.

The strengths of this study are its state-of-the art methods and its unrivalled sample size in researching multimorbidity and CKD. Observing similarities across two distinct cohorts does increase generalisability, but we did not expect results to be identical given differences in the frequency of blood tests, lifestyles, genetic backgrounds and variation of timely diagnoses of conditions such as pulmonary disease or heart failure, which can be challenging especially in inactive patients. For example, respiratory conditions were more common in SAIL than in SCREAM, which is consistent with the high rates of these conditions in Wales compared to Sweden [45]. Our analyses were restricted to participants whose eGFR crossed thresholds, and future work should consider clustering analyses in other populations, e.g. people with stable kidney function and people on dialysis. We openly provide the statistical code that we used for this work and encourage other researchers to replicate this analysis in their settings. Given age and kidney function are closely linked [46], the conditions prominent in each eGFR strata will have been largely influenced by age. It is unclear to what extent changes in clusters as eGFR declines are explained by advancing age rather than being specific to changes in kidney function. There are inherent limitations of health records research in that they rely on routine coding, a subjective process that if incomplete can lead to misclassification of clusters identified. We tried to improve the sensitivity of our ascertainment of chronic conditions by using previously validated algorithms [16], supplemented in some cases with medication data. We chose to enrich the definitions of depression and chronic pain with prescribing data, which will have increased the prevalence of these conditions and contributed to them featuring clusters. Other conditions may have featured more prominently in the clusters if we had used prescribing data to define them, also. We studied patients across the range of eGFR, without considering proteinuria data. Many of the patients in the eGFR categories 75 and 90 were therefore unlikely to have CKD, and instead, their inclusion allowed us to study clusters of conditions in people with good kidney function. Some of the follow-up period in SAIL was during the COVID-19 pandemic, when blood tests and recording of chronic conditions may have been inconsistent. However, this was a small proportion of the follow-up period and results were, overall, similar to SCREAM. We used k-modes as the clustering method, whereas other studies have used alternative techniques such as hierarchical clustering [47], latent class analysis [37] and consensus clustering [22]. This may limit our capacity to compare findings across studies. Finally, we did not account for the severity of chronic conditions. Such information may have been useful, but with a heterogenous list of conditions, this would have been challenging to ascertain for each condition or to include in the analysis.

## Conclusions

In summary, our study shows that there are clinically meaningful clusters of conditions which vary with declining kidney function. Cardiovascular conditions are prominent at low eGFR and associated with adverse outcomes, and hence, cardiovascular risk assessment and management should be included in the management of these patients. Importantly, chronic pain and depression are also common across the spectrum of kidney function but these conditions currently receive less attention or have fewer available treatment options in CKD. These data illustrate that CKD is not simply a biochemical ‘diagnosis’ but exists as part of the complex interactions between multiple chronic conditions. Identification and awareness of clusters of conditions may inform public health initiatives and permit health professionals to provide targeted interventions for patients with CKD.

## Supplementary Information

**Additional file 1:**

## Data Availability

The data that support the findings of this study are available from SCREAM and SAIL, for collaborative research projects subject to successful application processes and fulfilment of ethics and GDPR regulations. Further details can be found by contacting juan.jesus.carrero@ki.se for SCREAM and at www.saildatabank.com for SAIL.

## Abbreviations

ACM: All-cause mortality
AUC: Area under the receiver operating characteristic curve
CI: Confidence interval
CKD: Chronic kidney disease
eGFR: Estimated glomerular filtration rate
HR: Hazard ratio
IQI: Interquartile interval
MACE: Major adverse cardiovascular events
O/E: Observed/expected
SAIL: Secure Anonymised Information Linkage Databank
SCREAM: Stockholm Creatinine Measurements project
