# Supplementary material for: The presence and impact of multimorbidity clusters on adverse outcomes across the spectrum of kidney function
Source: BMC Med. 2022 Nov 1;20:420. doi: 10.1186/s12916-022-02628-2 (PMC9623942; doi:10.1186/s12916-022-02628-2)
Supplement: Supplementary file 1 — Additional file 1: [file 12916_2022_2628_MOESM1_ESM.docx]

**Additional File 1**

*Interpolation of kidney function*

We selected our cohorts through evaluation of linear trajectories of estimated glomerular filtration rate (eGFR) decline using all available creatinine measurements per participant. This aimed to reduce outcome misclassification bias owing to intrinsic eGFR variability, and to confirm whether eGFR declines were sustained over time. To that end, we fitted a linear mixed effects model with a random intercept and slope of time to interpolate the date at which each participant reached the following eGFR thresholds: 90, 75, 60, 45, 30 and 15 mL/min/1.73m^2^.^17^ The model included only time as a covariate, assuming linearity; furthermore, we assumed an unstructured variance-covariance matrix for the random effects. The model equation for the i^th^ participant is therefore:

$$eGFR_{i} =\beta_{0}+b_{0i} + \left( \beta_{1} +b_{1i} \right)time_{i}$$

Using the fitted model coefficients and the predicted random effects for each participant, we can estimate the time at which a given eGFR threshold (named (eGFR_T_) was reached using the following equation:

$$time_{i}=\frac{eGFR_{T}-\beta_{0}-b_{0i}}{\beta_{1}+b_{1i}}$$

The plot below illustrates the interpolation procedure in practice: according to our procedure, participant with ID = 1 will be included for the analysis at the eGFR = 60 and eGFR = 75 levels, while participant with ID = 2 will be included for the eGFR = 90 analysis, with corresponding index dates based on the results of the mixed modelling analysis (e.g., the subject-specific dashed lines).

We note that participants could be included in more than one eGFR category through their disease course and extent of follow up. In this design, participants undergoing long-term kidney replacement therapy were excluded after it began, as were participants with stable eGFR that did not cross any of the abovementioned thresholds.

Figure S1A. SCREAM participant inclusion flow chart. RRT, renal replacement therapy

Figure S1B. SAIL participant inclusion flow chart. RRT, renal replacement therapy

Table S1. ICD-10 codes for major adverse cardiovascular events (MACE)

| **Outcome** | **Diagnosis** | **ICD-10 codes** |
| --- | --- | --- |
| MACE3 | Cardiovascular death | Death attributed to G45‐G46, H341, I* |
|  | Hospitalisation attributed to myocardial infarction | I21, I22, I23 |
|  | Hospitalisation attributed to stroke | H341, G45, G46, I60, I61, I63, I64 |
| MACE4 | Cardiovascular death | Death attributed to G45‐G46, H341, I* |
|  | Hospitalisation attributed to myocardial infarction | I21, I22, I23 |
|  | Hospitalisation attributed to stroke | H341, G45, G46, I60, I61, I63, I64 |
|  | Hospitalisation attributed to heart failure | I099, I110, I130, I132, I255, I420, I425-429, I43, I50 |

Figure S2A. Comparison of SCREAM participants included and excluded from the analysis. A Birth dates; B Number of eGFR measurements; C Proportion female sex

Figure S2B. Comparison of SAIL participants included and excluded from the analysis. A Birth dates; B Number of eGFR measurements; C Proportion female sex

Figure S3A. Proportion of conditions by eGFR category in SCREAM

Figure S3B. Proportion of conditions by eGFR category in SAIL

Figure S4. Elbow plots by eGFR category. These plots help choose the optimal number of clusters. The optimal number of clusters is where the line flattens i.e. where increasing the number of clusters leads to an only marginal reduction in the within-cluster distance.

A. SCREAM

B. SAIL

Figure S5. Gradient plots derived from goodness of fit elbow plots by eGFR category. These plots are an alternative way to display the data in Figure S4, which may be simpler to interpret. The optimal number of clusters is where the line approaches 0.

A. SCREAM

B. SAIL

Table S2A. Condition prevalence by cluster in SCREAM

| eGFR | Condition | Cluster_1 | Cluster_2 | Cluster_3 | Cluster_4 | Cluster_5 | Cluster_6 | Cluster_7 | Cluster_8 | Cluster_9 |
| --- | --- | --- | --- | --- | --- | --- | --- | --- | --- | --- |
| 15 | n | 790 | 1,383 | 212 | 74 | 354 | 77 | 63 | NA | NA |
| 15 | Age | 75.4 | 70 | 77.06 | 73.34 | 70.23 | 78.91 | 74.1 | NA | NA |
| 15 | Gender = Female | 48.23% | 34.85% | 33.96% | 75.68% | 40.11% | 29.87% | 25.40% | NA | NA |
| 15 | Alcohol Misuse | 3.16% | 2.31% | 3.77% | 1.35% | 5.65% | 2.60% | 1.59% | NA | NA |
| 15 | Asthma | 7.09% | 1.52% | 4.25% | 9.46% | 3.67% | 5.19% | 1.59% | NA | NA |
| 15 | Atrial Fibrillation | 5.57% | 1.45% | 2.83% | 0.00% | 1.98% | 10.39% | 4.76% | NA | NA |
| 15 | Cancer | 10.38% | 12.15% | 17.92% | 14.86% | 16.67% | 64.94% | 4.76% | NA | NA |
| 15 | CKD | 94.81% | 81.49% | 89.62% | 91.89% | 86.16% | 97.40% | 95.24% | NA | NA |
| 15 | Chronic Pain | 86.96% | 0.00% | 68.87% | 90.54% | 100.00% | 28.57% | 0.00% | NA | NA |
| 15 | Chronic Viral Hepatitis B | 0.38% | 0.43% | 0.47% | 1.35% | 0.56% | 0.00% | 0.00% | NA | NA |
| 15 | Cirrhosis | 1.27% | 0.07% | 0.00% | 0.00% | 0.28% | 0.00% | 0.00% | NA | NA |
| 15 | Dementia | 2.91% | 3.90% | 8.02% | 0.00% | 2.26% | 2.60% | 4.76% | NA | NA |
| 15 | Depression | 11.77% | 6.94% | 10.85% | 18.92% | 13.56% | 7.79% | 11.11% | NA | NA |
| 15 | Diabetes | 76.58% | 19.74% | 59.43% | 5.41% | 0.00% | 84.42% | 66.67% | NA | NA |
| 15 | Epilepsy | 2.41% | 1.30% | 3.77% | 0.00% | 2.82% | 1.30% | 4.76% | NA | NA |
| 15 | Heart Failure | 65.95% | 14.61% | 0.00% | 9.46% | 0.00% | 90.91% | 34.92% | NA | NA |
| 15 | Hypertension | 88.61% | 69.78% | 90.09% | 85.14% | 73.16% | 92.21% | 88.89% | NA | NA |
| 15 | Hypothyroidism | 13.04% | 6.65% | 11.79% | 60.81% | 0.00% | 14.29% | 6.35% | NA | NA |
| 15 | IBD | 1.52% | 1.66% | 0.94% | 1.35% | 3.67% | 0.00% | 1.59% | NA | NA |
| 15 | IBS | 0.76% | 0.43% | 0.47% | 1.35% | 0.00% | 0.00% | 0.00% | NA | NA |
| 15 | Multiple Sclerosis | 0.25% | 0.07% | 0.00% | 0.00% | 0.28% | 0.00% | 0.00% | NA | NA |
| 15 | MI | 16.08% | 5.86% | 8.49% | 4.05% | 6.21% | 90.91% | 100.00% | NA | NA |
| 15 | Parkinson's Disease | 1.90% | 0.87% | 0.47% | 0.00% | 0.28% | 0.00% | 0.00% | NA | NA |
| 15 | Pulmonary Disease | 17.97% | 7.16% | 9.91% | 8.11% | 10.45% | 20.78% | 4.76% | NA | NA |
| 15 | PUD | 2.15% | 1.01% | 4.25% | 1.35% | 2.26% | 2.60% | 1.59% | NA | NA |
| 15 | PVD | 15.57% | 5.42% | 13.21% | 4.05% | 7.34% | 22.08% | 23.81% | NA | NA |
| 15 | Psoriasis | 5.57% | 2.39% | 4.25% | 2.70% | 6.21% | 3.90% | 1.59% | NA | NA |
| 15 | Rheumatoid Arthritis | 5.70% | 2.68% | 4.72% | 62.16% | 0.00% | 6.49% | 1.59% | NA | NA |
| 15 | Schizophrenia | 0.89% | 1.30% | 0.00% | 0.00% | 1.13% | 1.30% | 0.00% | NA | NA |
| 15 | Severe Constipation | 8.23% | 1.95% | 7.55% | 9.46% | 5.65% | 5.19% | 6.35% | NA | NA |
| 15 | Stroke | 17.22% | 9.91% | 100.00% | 4.05% | 0.00% | 11.69% | 71.43% | NA | NA |
| 30 | n | 2,582 | 3,455 | 150 | 3,259 | 1,338 | 453 | 967 | 314 | 303 |
| 30 | Age | 76.94 | 79.4 | 80.42 | 79.13 | 85.91 | 77.31 | 82.23 | 77.38 | 81.13 |
| 30 | Gender = Female | 47.79% | 48.05% | 64.00% | 53.30% | 59.42% | 43.71% | 50.78% | 57.64% | 35.64% |
| 30 | Alcohol Misuse | 3.18% | 3.24% | 5.33% | 2.45% | 2.62% | 1.77% | 3.00% | 7.96% | 2.31% |
| 30 | Asthma | 2.01% | 5.38% | 69.33% | 3.07% | 7.55% | 4.19% | 4.96% | 2.55% | 3.96% |
| 30 | Atrial Fibrillation | 0.93% | 4.95% | 11.33% | 2.79% | 9.79% | 1.55% | 6.62% | 6.69% | 5.61% |
| 30 | Cancer | 9.14% | 11.81% | 13.33% | 15.83% | 15.47% | 70.86% | 12.00% | 6.37% | 14.85% |
| 30 | CKD | 29.74% | 54.56% | 70.00% | 40.01% | 55.16% | 54.30% | 56.05% | 61.46% | 67.99% |
| 30 | Chronic Pain | 21.26% | 33.43% | 96.67% | 37.62% | 100.00% | 95.14% | 88.31% | 98.41% | 33.66% |
| 30 | Chronic Viral Hepatitis B | 0.12% | 0.23% | 0.67% | 0.12% | 0.15% | 0.44% | 0.21% | 0.64% | 0.00% |
| 30 | Cirrhosis | 0.54% | 0.93% | 0.00% | 0.21% | 0.67% | 0.66% | 0.41% | 1.27% | 0.00% |
| 30 | Dementia | 6.27% | 5.67% | 6.67% | 6.63% | 7.17% | 3.97% | 9.00% | 7.01% | 6.27% |
| 30 | Depression | 8.25% | 4.69% | 65.33% | 7.70% | 6.80% | 7.06% | 11.17% | 71.02% | 7.59% |
| 30 | Diabetes | 9.99% | 77.08% | 55.33% | 0.00% | 0.00% | 70.42% | 31.23% | 86.62% | 82.18% |
| 30 | Epilepsy | 1.51% | 1.19% | 2.67% | 1.66% | 1.79% | 1.99% | 4.45% | 2.87% | 0.66% |
| 30 | Heart Failure | 15.49% | 57.34% | 82.67% | 0.00% | 100.00% | 3.75% | 22.34% | 71.97% | 87.46% |
| 30 | Hypertension | 0.00% | 97.22% | 91.33% | 100.00% | 76.98% | 27.81% | 96.79% | 43.31% | 93.40% |
| 30 | Hypothyroidism | 6.24% | 11.78% | 14.67% | 9.91% | 13.75% | 9.49% | 12.10% | 12.42% | 11.55% |
| 30 | IBD | 1.36% | 1.04% | 3.33% | 1.20% | 1.12% | 0.88% | 1.14% | 1.91% | 0.99% |
| 30 | IBS | 0.23% | 0.43% | 2.00% | 0.43% | 1.12% | 0.00% | 0.52% | 1.27% | 0.33% |
| 30 | Multiple Sclerosis | 0.12% | 0.12% | 0.00% | 0.15% | 0.07% | 0.22% | 0.10% | 0.32% | 0.00% |
| 30 | MI | 6.74% | 12.42% | 76.00% | 3.68% | 17.34% | 6.84% | 54.29% | 9.87% | 66.01% |
| 30 | Parkinson's Disease | 1.12% | 0.81% | 1.33% | 0.80% | 1.05% | 0.88% | 0.72% | 1.91% | 0.99% |
| 30 | Pulmonary Disease | 7.59% | 14.18% | 40.00% | 6.72% | 20.48% | 7.73% | 13.34% | 17.83% | 21.78% |
| 30 | PUD | 1.12% | 1.68% | 2.67% | 1.53% | 3.51% | 0.44% | 2.28% | 3.50% | 4.62% |
| 30 | PVD | 3.64% | 7.87% | 16.00% | 6.01% | 9.49% | 4.42% | 11.79% | 12.74% | 86.47% |
| 30 | Psoriasis | 3.02% | 3.82% | 3.33% | 3.59% | 4.71% | 4.19% | 3.72% | 4.78% | 3.30% |
| 30 | Rheumatoid Arthritis | 4.69% | 5.35% | 13.33% | 5.86% | 10.09% | 8.61% | 7.34% | 8.28% | 4.62% |
| 30 | Schizophrenia | 1.39% | 0.52% | 0.00% | 0.43% | 0.15% | 1.10% | 0.10% | 0.64% | 0.00% |
| 30 | Severe Constipation | 3.72% | 5.35% | 15.33% | 4.36% | 10.76% | 2.43% | 8.27% | 11.46% | 8.91% |
| 30 | Stroke | 12.01% | 15.28% | 16.67% | 12.18% | 19.58% | 10.82% | 82.01% | 17.20% | 67.00% |
| 45 | n | 13,421 | 4,837 | 3,854 | 7,426 | 5,897 | 1,576 | 152 | NA | NA |
| 45 | Age | 77.67 | 82.96 | 82.85 | 80.12 | 78.21 | 82 | 81.7 | NA | NA |
| 45 | Gender = Female | 50.12% | 59.95% | 53.87% | 68.07% | 49.26% | 48.67% | 44.74% | NA | NA |
| 45 | Alcohol Misuse | 2.85% | 3.18% | 2.57% | 2.18% | 2.31% | 3.05% | 1.97% | NA | NA |
| 45 | Asthma | 3.76% | 9.63% | 4.13% | 4.59% | 2.07% | 8.25% | 7.24% | NA | NA |
| 45 | Atrial Fibrillation | 2.24% | 7.90% | 3.79% | 3.22% | 0.76% | 8.12% | 100.00% | NA | NA |
| 45 | Cancer | 12.47% | 15.71% | 13.31% | 15.62% | 11.85% | 13.20% | 19.74% | NA | NA |
| 45 | CKD | 18.33% | 25.22% | 15.10% | 15.46% | 10.21% | 27.28% | 30.26% | NA | NA |
| 45 | Chronic Pain | 23.42% | 78.97% | 36.30% | 100.00% | 0.00% | 79.06% | 0.00% | NA | NA |
| 45 | Chronic Viral Hepatitis B | 0.14% | 0.14% | 0.03% | 0.12% | 0.07% | 0.38% | 0.00% | NA | NA |
| 45 | Cirrhosis | 0.76% | 0.33% | 0.13% | 0.54% | 0.53% | 0.44% | 0.66% | NA | NA |
| 45 | Dementia | 5.15% | 7.26% | 10.51% | 6.29% | 6.26% | 5.33% | 3.95% | NA | NA |
| 45 | Depression | 7.74% | 10.92% | 11.57% | 12.44% | 5.78% | 10.91% | 9.21% | NA | NA |
| 45 | Diabetes | 60.00% | 25.26% | 0.00% | 0.00% | 0.00% | 32.93% | 14.47% | NA | NA |
| 45 | Epilepsy | 1.27% | 1.96% | 3.68% | 1.28% | 0.70% | 2.22% | 1.97% | NA | NA |
| 45 | Heart Failure | 9.74% | 100.00% | 18.06% | 0.00% | 15.30% | 100.00% | 89.47% | NA | NA |
| 45 | Hypertension | 86.60% | 81.06% | 76.05% | 64.06% | 0.00% | 84.71% | 94.74% | NA | NA |
| 45 | Hypothyroidism | 8.52% | 13.19% | 10.92% | 11.61% | 5.10% | 13.77% | 9.87% | NA | NA |
| 45 | IBD | 1.01% | 0.87% | 0.78% | 1.39% | 1.05% | 0.76% | 0.00% | NA | NA |
| 45 | IBS | 0.29% | 0.91% | 0.42% | 0.61% | 0.24% | 0.76% | 0.00% | NA | NA |
| 45 | Multiple Sclerosis | 0.14% | 0.19% | 0.08% | 0.15% | 0.12% | 0.19% | 0.00% | NA | NA |
| 45 | MI | 10.02% | 0.00% | 11.16% | 5.91% | 5.56% | 100.00% | 51.97% | NA | NA |
| 45 | Parkinson's Disease | 0.65% | 1.24% | 1.61% | 0.93% | 0.95% | 1.02% | 0.66% | NA | NA |
| 45 | Pulmonary Disease | 7.21% | 20.26% | 8.93% | 8.34% | 6.10% | 21.83% | 19.74% | NA | NA |
| 45 | PUD | 1.11% | 1.94% | 1.48% | 1.16% | 0.64% | 2.54% | 3.95% | NA | NA |
| 45 | PVD | 6.90% | 9.10% | 6.49% | 4.71% | 2.31% | 14.53% | 9.87% | NA | NA |
| 45 | Psoriasis | 4.03% | 4.47% | 3.76% | 4.77% | 2.36% | 4.70% | 1.32% | NA | NA |
| 45 | Rheumatoid Arthritis | 4.00% | 8.17% | 5.60% | 8.40% | 2.78% | 10.53% | 5.92% | NA | NA |
| 45 | Schizophrenia | 0.60% | 0.29% | 0.29% | 0.54% | 0.93% | 0.32% | 0.66% | NA | NA |
| 45 | Severe Constipation | 3.32% | 9.49% | 5.94% | 5.17% | 1.95% | 9.07% | 8.55% | NA | NA |
| 45 | Stroke | 10.80% | 17.92% | 100.00% | 0.00% | 0.00% | 21.32% | 8.55% | NA | NA |
| 60 | n | 26,463 | 22,991 | 4,667 | 2,151 | 22,182 | 959 | NA | NA | NA |
| 60 | Age | 76.13 | 76.21 | 79.57 | 76.22 | 74.28 | 75.35 | NA | NA | NA |
| 60 | Gender = Female | 64.22% | 49.50% | 57.62% | 82.29% | 49.00% | 37.23% | NA | NA | NA |
| 60 | Alcohol Misuse | 2.96% | 2.93% | 3.77% | 1.81% | 1.94% | 5.32% | NA | NA | NA |
| 60 | Asthma | 5.13% | 2.84% | 6.73% | 5.11% | 1.50% | 5.32% | NA | NA | NA |
| 60 | Atrial Fibrillation | 3.38% | 2.34% | 5.49% | 2.05% | 0.89% | 3.65% | NA | NA | NA |
| 60 | Cancer | 14.03% | 11.57% | 13.09% | 12.74% | 9.76% | 11.68% | NA | NA | NA |
| 60 | CKD | 8.92% | 6.60% | 10.56% | 6.56% | 4.52% | 15.02% | NA | NA | NA |
| 60 | Chronic Pain | 100.00% | 0.00% | 100.00% | 46.63% | 0.00% | 61.21% | NA | NA | NA |
| 60 | Chronic Viral Hepatitis B | 0.14% | 0.05% | 0.11% | 0.00% | 0.07% | 0.21% | NA | NA | NA |
| 60 | Cirrhosis | 0.46% | 0.34% | 0.28% | 0.19% | 0.38% | 0.31% | NA | NA | NA |
| 60 | Dementia | 4.18% | 4.15% | 7.67% | 6.32% | 4.01% | 5.01% | NA | NA | NA |
| 60 | Depression | 12.59% | 6.53% | 15.92% | 15.02% | 6.10% | 11.37% | NA | NA | NA |
| 60 | Diabetes | 19.91% | 23.84% | 23.27% | 11.90% | 9.94% | 100.00% | NA | NA | NA |
| 60 | Epilepsy | 1.00% | 1.44% | 4.07% | 1.07% | 0.82% | 2.40% | NA | NA | NA |
| 60 | Heart Failure | 14.63% | 13.56% | 24.49% | 13.02% | 8.10% | 33.47% | NA | NA | NA |
| 60 | Hypertension | 60.37% | 100.00% | 75.66% | 0.00% | 0.00% | 91.14% | NA | NA | NA |
| 60 | Hypothyroidism | 6.92% | 7.88% | 12.19% | 100.00% | 0.00% | 8.76% | NA | NA | NA |
| 60 | IBD | 1.18% | 0.68% | 0.99% | 1.02% | 0.83% | 0.73% | NA | NA | NA |
| 60 | IBS | 0.79% | 0.34% | 0.90% | 0.65% | 0.27% | 0.52% | NA | NA | NA |
| 60 | Multiple Sclerosis | 0.20% | 0.10% | 0.17% | 0.23% | 0.17% | 0.00% | NA | NA | NA |
| 60 | MI | 7.47% | 9.31% | 12.41% | 4.56% | 4.04% | 22.11% | NA | NA | NA |
| 60 | Parkinson's Disease | 0.91% | 0.67% | 1.48% | 1.21% | 0.69% | 0.63% | NA | NA | NA |
| 60 | Pulmonary Disease | 9.27% | 6.34% | 11.33% | 8.32% | 4.00% | 15.85% | NA | NA | NA |
| 60 | PUD | 0.82% | 0.67% | 1.44% | 0.60% | 0.39% | 1.67% | NA | NA | NA |
| 60 | PVD | 2.82% | 2.45% | 4.78% | 2.14% | 1.47% | 100.00% | NA | NA | NA |
| 60 | Psoriasis | 4.85% | 3.60% | 4.97% | 3.81% | 2.67% | 6.99% | NA | NA | NA |
| 60 | Rheumatoid Arthritis | 7.15% | 2.83% | 7.91% | 6.09% | 2.23% | 5.21% | NA | NA | NA |
| 60 | Schizophrenia | 0.45% | 0.37% | 0.41% | 1.67% | 0.91% | 0.52% | NA | NA | NA |
| 60 | Severe Constipation | 4.64% | 2.40% | 8.29% | 5.67% | 1.73% | 4.69% | NA | NA | NA |
| 60 | Stroke | 0.00% | 16.06% | 100.00% | 3.77% | 6.40% | 24.61% | NA | NA | NA |
| 75 | n | 42,753 | 32,312 | 2,446 | 5,961 | 65,668 | 5,187 | NA | NA | NA |
| 75 | Age | 68.02 | 70.92 | 73.1 | 75.32 | 65.06 | 71.6 | NA | NA | NA |
| 75 | Gender = Female | 64.92% | 47.09% | 41.17% | 53.04% | 50.51% | 58.07% | NA | NA | NA |
| 75 | Alcohol Misuse | 3.08% | 2.74% | 3.64% | 6.79% | 1.60% | 2.95% | NA | NA | NA |
| 75 | Asthma | 3.81% | 2.28% | 2.04% | 7.87% | 1.01% | 5.24% | NA | NA | NA |
| 75 | Atrial Fibrillation | 1.45% | 1.55% | 1.47% | 5.96% | 0.43% | 2.12% | NA | NA | NA |
| 75 | Cancer | 0.00% | 10.19% | 8.87% | 12.40% | 7.16% | 100.00% | NA | NA | NA |
| 75 | CKD | 4.10% | 3.83% | 4.13% | 9.08% | 2.25% | 7.00% | NA | NA | NA |
| 75 | Chronic Pain | 100.00% | 0.00% | 0.00% | 92.28% | 0.00% | 100.00% | NA | NA | NA |
| 75 | Chronic Viral Hepatitis B | 0.10% | 0.06% | 0.08% | 0.13% | 0.05% | 0.10% | NA | NA | NA |
| 75 | Cirrhosis | 0.29% | 0.23% | 0.29% | 0.47% | 0.21% | 0.29% | NA | NA | NA |
| 75 | Dementia | 2.29% | 2.28% | 5.85% | 5.69% | 1.49% | 2.06% | NA | NA | NA |
| 75 | Depression | 14.06% | 6.00% | 8.99% | 16.32% | 5.82% | 14.27% | NA | NA | NA |
| 75 | Diabetes | 13.08% | 20.70% | 9.48% | 30.28% | 5.96% | 12.80% | NA | NA | NA |
| 75 | Epilepsy | 1.09% | 1.23% | 5.11% | 4.55% | 0.50% | 1.06% | NA | NA | NA |
| 75 | Heart Failure | 2.08% | 5.77% | 9.93% | 53.92% | 2.33% | 2.53% | NA | NA | NA |
| 75 | Hypertension | 36.88% | 100.00% | 0.00% | 97.11% | 0.00% | 41.97% | NA | NA | NA |
| 75 | Hypothyroidism | 7.77% | 6.15% | 5.23% | 10.80% | 3.69% | 9.08% | NA | NA | NA |
| 75 | IBD | 1.12% | 0.65% | 0.86% | 1.06% | 0.85% | 1.06% | NA | NA | NA |
| 75 | IBS | 0.90% | 0.29% | 0.20% | 0.79% | 0.27% | 0.60% | NA | NA | NA |
| 75 | Multiple Sclerosis | 0.37% | 0.13% | 0.33% | 0.17% | 0.19% | 0.29% | NA | NA | NA |
| 75 | MI | 3.58% | 6.66% | 5.15% | 16.79% | 1.98% | 3.72% | NA | NA | NA |
| 75 | Parkinson's Disease | 0.93% | 0.60% | 0.98% | 1.61% | 0.47% | 0.79% | NA | NA | NA |
| 75 | Pulmonary Disease | 5.50% | 4.51% | 5.40% | 15.70% | 2.11% | 6.96% | NA | NA | NA |
| 75 | PUD | 0.58% | 0.46% | 0.78% | 1.80% | 0.21% | 0.67% | NA | NA | NA |
| 75 | PVD | 2.18% | 2.42% | 2.45% | 7.62% | 0.59% | 2.35% | NA | NA | NA |
| 75 | Psoriasis | 4.43% | 3.44% | 2.74% | 5.40% | 2.64% | 4.67% | NA | NA | NA |
| 75 | Rheumatoid Arthritis | 4.87% | 1.98% | 2.33% | 6.88% | 1.50% | 4.97% | NA | NA | NA |
| 75 | Schizophrenia | 0.58% | 0.39% | 0.70% | 0.42% | 0.79% | 0.25% | NA | NA | NA |
| 75 | Severe Constipation | 3.03% | 1.59% | 2.41% | 7.92% | 0.91% | 1.95% | NA | NA | NA |
| 75 | Stroke | 2.92% | 10.67% | 100.00% | 66.00% | 0.00% | 3.74% | NA | NA | NA |
| 90 | n | 26,586 | 1,440 | 165,836 | 2,503 | 14,681 | NA | NA | NA | NA |
| 90 | Age | 63.3 | 67.25 | 55.59 | 66.72 | 63.47 | NA | NA | NA | NA |
| 90 | Gender = Female | 43.96% | 41.94% | 51.72% | 60.85% | 57.09% | NA | NA | NA | NA |
| 90 | Alcohol Misuse | 4.15% | 7.92% | 2.47% | 13.66% | 5.50% | NA | NA | NA | NA |
| 90 | Asthma | 1.82% | 6.25% | 1.20% | 17.18% | 4.27% | NA | NA | NA | NA |
| 90 | Atrial Fibrillation | 0.67% | 2.01% | 0.20% | 2.08% | 1.55% | NA | NA | NA | NA |
| 90 | Cancer | 7.06% | 59.79% | 4.81% | 10.91% | 6.38% | NA | NA | NA | NA |
| 90 | CKD | 2.58% | 7.92% | 1.95% | 6.15% | 4.27% | NA | NA | NA | NA |
| 90 | Chronic Pain | 0.00% | 95.49% | 26.66% | 100.00% | 100.00% | NA | NA | NA | NA |
| 90 | Chronic Viral Hepatitis B | 0.06% | 0.35% | 0.08% | 0.36% | 0.16% | NA | NA | NA | NA |
| 90 | Cirrhosis | 0.21% | 0.76% | 0.14% | 0.72% | 0.32% | NA | NA | NA | NA |
| 90 | Dementia | 0.75% | 2.57% | 0.39% | 2.20% | 0.91% | NA | NA | NA | NA |
| 90 | Depression | 6.94% | 17.36% | 9.30% | 22.13% | 15.64% | NA | NA | NA | NA |
| 90 | Diabetes | 20.24% | 85.49% | 4.65% | 16.78% | 20.69% | NA | NA | NA | NA |
| 90 | Epilepsy | 1.37% | 6.32% | 0.82% | 3.16% | 1.68% | NA | NA | NA | NA |
| 90 | Heart Failure | 3.41% | 11.04% | 0.88% | 12.74% | 4.24% | NA | NA | NA | NA |
| 90 | Hypertension | 100.00% | 73.12% | 0.00% | 45.11% | 100.00% | NA | NA | NA | NA |
| 90 | Hypothyroidism | 4.68% | 7.71% | 3.40% | 8.35% | 7.43% | NA | NA | NA | NA |
| 90 | IBD | 0.76% | 1.39% | 1.06% | 2.12% | 1.16% | NA | NA | NA | NA |
| 90 | IBS | 0.27% | 0.83% | 0.49% | 1.00% | 1.03% | NA | NA | NA | NA |
| 90 | Multiple Sclerosis | 0.20% | 0.28% | 0.36% | 0.44% | 0.46% | NA | NA | NA | NA |
| 90 | MI | 5.08% | 9.51% | 1.11% | 7.03% | 5.42% | NA | NA | NA | NA |
| 90 | Parkinson's Disease | 0.30% | 1.39% | 0.33% | 0.84% | 0.73% | NA | NA | NA | NA |
| 90 | Pulmonary Disease | 3.54% | 8.61% | 0.89% | 100.00% | 0.00% | NA | NA | NA | NA |
| 90 | PUD | 0.36% | 1.18% | 0.21% | 1.08% | 0.87% | NA | NA | NA | NA |
| 90 | PVD | 1.63% | 5.62% | 0.41% | 5.07% | 2.38% | NA | NA | NA | NA |
| 90 | Psoriasis | 3.83% | 5.62% | 2.77% | 7.11% | 5.27% | NA | NA | NA | NA |
| 90 | Rheumatoid Arthritis | 1.19% | 3.33% | 1.47% | 5.43% | 3.98% | NA | NA | NA | NA |
| 90 | Schizophrenia | 0.39% | 1.04% | 0.72% | 1.32% | 0.46% | NA | NA | NA | NA |
| 90 | Severe Constipation | 1.00% | 3.75% | 0.89% | 5.95% | 2.55% | NA | NA | NA | NA |
| 90 | Stroke | 8.04% | 62.92% | 1.86% | 8.15% | 6.64% | NA | NA | NA | NA |

Table S2B. Condition prevalence by cluster in SAIL

| eGFR | Condition | Cluster_1 | Cluster_2 | Cluster_3 | Cluster_4 | Cluster_5 | Cluster_6 | Cluster_7 | Cluster_8 |
| --- | --- | --- | --- | --- | --- | --- | --- | --- | --- |
| 15 | n | 1,656 | 452 | 294 | 357 | 608 | 289 | 159 | 519 |
| 15 | Age | 72.74 | 78.06 | 79.02 | 78.9 | 67.14 | 72.88 | 75.43 | 72.82 |
| 15 | GENDER = Female | 46.07% | 33.85% | 42.52% | 35.85% | 50.66% | 42.91% | 49.06% | 36.80% |
| 15 | Alcohol Misuse | 2.29% | 2.43% | 2.72% | 1.96% | 5.59% | 3.81% | <3% | 1.54% |
| 15 | Asthma | 13.59% | 10.84% | 26.87% | 13.73% | 14.97% | 18.34% | 15.09% | 8.48% |
| 15 | Atrial Fibrillation | 11.29% | 18.36% | 73.47% | 17.93% | 11.51% | 9.00% | 12.58% | 9.44% |
| 15 | Cancer | 10.75% | 100.00% | 21.77% | 100.00% | 0.00% | 15.22% | 12.58% | 0.00% |
| 15 | Heart Failure | 13.29% | 19.91% | 91.84% | 14.57% | 18.42% | 14.53% | 84.91% | 6.55% |
| 15 | Chronic Pain | 55.50% | 29.87% | 59.18% | 0.00% | 22.86% | 70.93% | 57.23% | 0.00% |
| 15 | Pulmonary Disease | 15.22% | 14.38% | 63.61% | 17.93% | 14.64% | 14.53% | 11.32% | 8.86% |
| 15 | Chronic Viral Hepatitis B | <3% | 0.00% | <3% | 0.00% | 0.00% | 0.00% | 0.00% | 0.00% |
| 15 | Cirrhosis | 0.91% | <3% | <3% | <3% | <3% | <3% | <3% | 1.73% |
| 15 | Dementia | 3.68% | 3.32% | 4.42% | 4.48% | 6.09% | 8.30% | 9.43% | 1.93% |
| 15 | Depression | 14.61% | 23.23% | 22.45% | 19.33% | 100.00% | 33.56% | 35.22% | 0.00% |
| 15 | Diabetes | 14.49% | 100.00% | 90.14% | 0.00% | 67.27% | 100.00% | 86.79% | 100.00% |
| 15 | Epilepsy | 2.17% | <3% | <3% | <3% | 2.30% | 4.50% | 5.66% | 1.54% |
| 15 | Hypertension | 83.94% | 86.73% | 92.52% | 80.39% | 85.69% | 88.58% | 91.82% | 89.98% |
| 15 | Hypothyroidism | 13.53% | 14.38% | 15.65% | 12.61% | 15.30% | 11.42% | 15.09% | 14.07% |
| 15 | IBD | 2.36% | 1.55% | 2.72% | 1.96% | 1.97% | <3% | 3.77% | 1.35% |
| 15 | IBS | 1.57% | 2.21% | <3% | 1.68% | 1.64% | 2.77% | <3% | 1.16% |
| 15 | Multiple Sclerosis | <3% | 0.00% | 0.00% | 0.00% | <3% | 0.00% | <3% | <3% |
| 15 | MI | 14.07% | 17.92% | 36.05% | 16.53% | 14.47% | 21.11% | 87.42% | 11.37% |
| 15 | Parkinson's Disease | 0.91% | <3% | <3% | <3% | <3% | <3% | <3% | <3% |
| 15 | PUD | 3.56% | 3.98% | 7.82% | 3.36% | 3.78% | 6.92% | 7.55% | 4.05% |
| 15 | PVD | 13.41% | 15.93% | 21.09% | 12.04% | 13.98% | 18.34% | 21.38% | 16.38% |
| 15 | Psoriasis | 2.17% | 2.88% | 3.74% | 3.08% | 2.30% | 2.77% | 3.77% | <3% |
| 15 | Rheumatoid Arthritis | 4.59% | 2.88% | 4.42% | 2.24% | 2.47% | 4.50% | 5.03% | 1.54% |
| 15 | Schizophrenia | 0.66% | 0.00% | <3% | <3% | 1.81% | 2.42% | 0.00% | <3% |
| 15 | Severe Constipation | 14.61% | 10.18% | 24.15% | 10.08% | 11.18% | 100.00% | 10.06% | 0.00% |
| 15 | Stroke | 13.16% | 11.73% | 14.97% | 16.81% | 16.78% | 20.42% | 71.70% | 11.56% |
| 30 | n | 3,212 | 5,197 | 5,452 | 7,065 | 2,614 | 872 | NA | NA |
| 30 | Age | 80.77 | 78.43 | 76.22 | 77.79 | 80.65 | 81.02 | NA | NA |
| 30 | GENDER = Female | 50.34% | 64.08% | 42.90% | 49.60% | 52.68% | 61.35% | NA | NA |
| 30 | Alcohol Misuse | 2.05% | 2.21% | 2.20% | 2.01% | 2.45% | 3.90% | NA | NA |
| 30 | Asthma | 17.75% | 15.39% | 11.67% | 10.54% | 18.67% | 15.94% | NA | NA |
| 30 | Atrial Fibrillation | 14.32% | 4.62% | 11.46% | 15.47% | 80.64% | 50.92% | NA | NA |
| 30 | Cancer | 25.06% | 25.51% | 22.76% | 25.24% | 25.40% | 27.64% | NA | NA |
| 30 | Heart Failure | 69.15% | 0.00% | 9.57% | 12.89% | 83.24% | 10.67% | NA | NA |
| 30 | Chronic Pain | 70.05% | 100.00% | 0.00% | 0.00% | 73.95% | 88.76% | NA | NA |
| 30 | Pulmonary Disease | 26.46% | 17.11% | 14.82% | 14.55% | 27.77% | 21.90% | NA | NA |
| 30 | Chronic Viral Hepatitis B | <3% | 0.00% | 0.00% | <3% | <3% | 0.00% | NA | NA |
| 30 | Cirrhosis | 0.78% | 1.12% | 1.23% | 0.69% | 1.34% | <3% | NA | NA |
| 30 | Dementia | 5.17% | 6.41% | 4.90% | 4.87% | 5.59% | 16.86% | NA | NA |
| 30 | Depression | 26.40% | 26.67% | 21.99% | 17.75% | 27.70% | 90.02% | NA | NA |
| 30 | Diabetes | 41.72% | 44.33% | 100.00% | 0.00% | 86.53% | 34.86% | NA | NA |
| 30 | Epilepsy | 1.74% | 1.46% | 1.17% | 1.57% | 1.38% | 5.28% | NA | NA |
| 30 | Hypertension | 84.87% | 81.95% | 84.26% | 76.53% | 85.27% | 86.70% | NA | NA |
| 30 | Hypothyroidism | 17.90% | 14.57% | 12.97% | 12.67% | 18.17% | 17.78% | NA | NA |
| 30 | IBD | 1.99% | 2.41% | 1.80% | 2.17% | 1.72% | 2.06% | NA | NA |
| 30 | IBS | 2.12% | 2.33% | 0.92% | 1.22% | 1.80% | 2.75% | NA | NA |
| 30 | Multiple Sclerosis | <3% | <3% | 0.18% | <3% | <3% | <3% | NA | NA |
| 30 | MI | 87.30% | 0.00% | 15.06% | 9.77% | 25.02% | 20.64% | NA | NA |
| 30 | Parkinson's Disease | 1.25% | 1.17% | 0.99% | 0.61% | 1.22% | 2.29% | NA | NA |
| 30 | PUD | 6.20% | 4.58% | 4.38% | 3.75% | 5.62% | 8.83% | NA | NA |
| 30 | PVD | 20.36% | 15.07% | 15.17% | 12.61% | 19.66% | 17.66% | NA | NA |
| 30 | Psoriasis | 1.31% | 1.69% | 1.43% | 1.19% | 1.95% | 2.75% | NA | NA |
| 30 | Rheumatoid Arthritis | 5.54% | 6.16% | 2.16% | 3.01% | 5.13% | 5.73% | NA | NA |
| 30 | Schizophrenia | 0.47% | 1.06% | 1.01% | 1.13% | 0.69% | 0.92% | NA | NA |
| 30 | Severe Constipation | 23.29% | 21.80% | 10.18% | 9.94% | 22.49% | 31.54% | NA | NA |
| 30 | Stroke | 15.35% | 9.76% | 15.15% | 13.45% | 18.13% | 86.93% | NA | NA |
| 45 | n | 10,056 | 40,000 | 6,990 | 5,391 | 3,123 | 2,476 | NA | NA |
| 45 | Age | 78.52 | 76.96 | 77.56 | 77.3 | 81.14 | 80.29 | NA | NA |
| 45 | GENDER = Female | 54.62% | 52.65% | 58.33% | 52.49% | 62.02% | 68.05% | NA | NA |
| 45 | Alcohol Misuse | 2.00% | 1.81% | 3.03% | 3.36% | 2.56% | 3.47% | NA | NA |
| 45 | Asthma | 9.95% | 5.15% | 81.97% | 7.12% | 3.14% | 15.11% | NA | NA |
| 45 | Atrial Fibrillation | 14.86% | 12.07% | 18.76% | 67.95% | 14.86% | 14.14% | NA | NA |
| 45 | Cancer | 68.07% | 14.62% | 12.29% | 13.69% | 10.12% | 20.96% | NA | NA |
| 45 | Heart Failure | 11.51% | 9.46% | 20.99% | 64.51% | 7.30% | 54.36% | NA | NA |
| 45 | Chronic Pain | 77.83% | 14.29% | 68.45% | 68.24% | 82.64% | 93.70% | NA | NA |
| 45 | Pulmonary Disease | 13.47% | 7.49% | 83.56% | 13.93% | 6.05% | 21.73% | NA | NA |
| 45 | Chronic Viral Hepatitis B | <3% | 0.02% | <3% | <3% | 0.00% | 0.00% | NA | NA |
| 45 | Cirrhosis | 0.98% | 0.84% | 1.20% | 1.67% | 0.93% | 1.29% | NA | NA |
| 45 | Dementia | 4.70% | 3.83% | 4.38% | 5.90% | 10.05% | 13.69% | NA | NA |
| 45 | Depression | 21.95% | 16.40% | 27.80% | 58.12% | 19.12% | 81.79% | NA | NA |
| 45 | Diabetes | 32.62% | 36.11% | 43.30% | 85.33% | 33.53% | 32.11% | NA | NA |
| 45 | Epilepsy | 1.65% | 1.17% | 1.49% | 1.58% | 3.97% | 3.15% | NA | NA |
| 45 | Hypertension | 33.51% | 81.48% | 87.91% | 90.54% | 94.72% | 81.30% | NA | NA |
| 45 | Hypothyroidism | 13.07% | 12.13% | 15.89% | 15.62% | 15.08% | 17.37% | NA | NA |
| 45 | IBD | 2.71% | 1.70% | 3.15% | 1.95% | 1.92% | 2.42% | NA | NA |
| 45 | IBS | 2.23% | 1.23% | 2.78% | 2.30% | 2.72% | 5.86% | NA | NA |
| 45 | Multiple Sclerosis | 0.18% | 0.09% | 0.10% | 0.13% | 0.19% | <3% | NA | NA |
| 45 | MI | 15.54% | 15.00% | 23.75% | 33.67% | 18.38% | 28.88% | NA | NA |
| 45 | Parkinson's Disease | 1.09% | 0.82% | 1.04% | 1.34% | 2.08% | 2.58% | NA | NA |
| 45 | PUD | 3.96% | 3.12% | 5.71% | 5.40% | 6.12% | 6.87% | NA | NA |
| 45 | PVD | 13.11% | 11.53% | 16.19% | 19.33% | 14.47% | 15.63% | NA | NA |
| 45 | Psoriasis | 1.58% | 1.16% | 1.87% | 1.84% | 1.44% | 1.62% | NA | NA |
| 45 | Rheumatoid Arthritis | 4.95% | 3.05% | 6.60% | 4.47% | 5.73% | 7.84% | NA | NA |
| 45 | Schizophrenia | 1.01% | 1.08% | 0.80% | 0.82% | 1.12% | 2.87% | NA | NA |
| 45 | Severe Constipation | 12.24% | 5.83% | 15.84% | 9.59% | 72.53% | 89.22% | NA | NA |
| 45 | Stroke | 11.36% | 9.44% | 15.45% | 20.22% | 70.73% | 8.20% | NA | NA |
| 60 | n | 29,274 | 12,383 | 66,079 | 8,486 | 7,918 | 2,310 | 2,724 | NA |
| 60 | Age | 75.64 | 74.19 | 73.04 | 68.68 | 70.49 | 73.62 | 77.94 | NA |
| 60 | GENDER = Female | 63.18% | 51.51% | 49.74% | 71.02% | 56.11% | 62.25% | 36.67% | NA |
| 60 | Alcohol Misuse | 2.06% | 3.31% | 1.72% | 4.96% | 4.53% | 6.54% | 3.67% | NA |
| 60 | Asthma | 7.39% | 78.13% | 4.84% | 14.44% | 6.02% | 30.78% | 9.21% | NA |
| 60 | Atrial Fibrillation | 10.51% | 14.90% | 8.89% | 6.92% | 12.00% | 13.98% | 100.00% | NA |
| 60 | Cancer | 20.27% | 19.97% | 18.07% | 17.55% | 19.02% | 20.65% | 23.09% | NA |
| 60 | Heart Failure | 9.22% | 17.39% | 5.87% | 5.94% | 11.35% | 15.67% | 42.58% | NA |
| 60 | Chronic Pain | 100.00% | 55.29% | 0.00% | 50.34% | 36.95% | 28.23% | 37.37% | NA |
| 60 | Pulmonary Disease | 6.01% | 79.59% | 4.67% | 10.08% | 5.99% | 100.00% | 13.51% | NA |
| 60 | Chronic Viral Hepatitis B | 0.03% | 0.06% | 0.02% | <3% | <3% | 0.00% | <3% | NA |
| 60 | Cirrhosis | 0.89% | 1.49% | 0.73% | 1.37% | 2.36% | 0.91% | 0.88% | NA |
| 60 | Dementia | 3.70% | 2.72% | 2.31% | 5.72% | 6.37% | 6.58% | 4.33% | NA |
| 60 | Depression | 15.18% | 23.09% | 7.75% | 100.00% | 100.00% | 100.00% | 11.67% | NA |
| 60 | Diabetes | 24.48% | 65.73% | 23.75% | 12.02% | 100.00% | 0.00% | 31.20% | NA |
| 60 | Epilepsy | 1.63% | 2.04% | 1.17% | 2.20% | 2.05% | 2.21% | 1.98% | NA |
| 60 | Hypertension | 75.97% | 77.91% | 66.02% | 0.00% | 83.25% | 71.86% | 80.91% | NA |
| 60 | Hypothyroidism | 13.16% | 13.17% | 10.06% | 15.61% | 14.40% | 14.68% | 13.00% | NA |
| 60 | IBD | 2.06% | 2.75% | 1.60% | 2.73% | 2.11% | 3.12% | 2.09% | NA |
| 60 | IBS | 2.81% | 2.92% | 1.22% | 3.72% | 3.31% | 3.64% | 1.76% | NA |
| 60 | Multiple Sclerosis | 0.24% | 0.15% | 0.12% | 0.49% | 0.28% | 0.30% | <3% | NA |
| 60 | MI | 12.48% | 20.24% | 9.37% | 7.71% | 17.02% | 18.05% | 100.00% | NA |
| 60 | Parkinson's Disease | 1.20% | 0.84% | 0.71% | 1.63% | 1.62% | 1.47% | 1.21% | NA |
| 60 | PUD | 3.96% | 4.85% | 2.21% | 3.25% | 3.95% | 4.94% | 4.26% | NA |
| 60 | PVD | 11.41% | 13.71% | 8.14% | 7.54% | 11.87% | 14.24% | 15.09% | NA |
| 60 | Psoriasis | 1.43% | 2.05% | 0.97% | 1.46% | 2.18% | 1.56% | 1.28% | NA |
| 60 | Rheumatoid Arthritis | 6.53% | 5.85% | 2.24% | 4.38% | 3.33% | 4.81% | 4.30% | NA |
| 60 | Schizophrenia | 0.64% | 0.76% | 0.66% | 4.74% | 2.98% | 2.86% | 0.37% | NA |
| 60 | Severe Constipation | 18.01% | 16.54% | 6.03% | 16.23% | 14.57% | 17.19% | 15.57% | NA |
| 60 | Stroke | 11.97% | 13.13% | 8.48% | 7.60% | 14.86% | 16.06% | 20.19% | NA |
| 75 | n | 95,957 | 84,041 | 3,754 | 9,023 | 11,278 | NA | NA | NA |
| 75 | Age | 68.55 | 61.65 | 73.4 | 69.02 | 67.36 | NA | NA | NA |
| 75 | GENDER = Female | 51.70% | 56.60% | 47.79% | 58.86% | 56.13% | NA | NA | NA |
| 75 | Alcohol Misuse | 3.22% | 2.94% | 5.57% | 6.01% | 2.46% | NA | NA | NA |
| 75 | Asthma | 9.40% | 9.72% | 39.61% | 86.68% | 7.97% | NA | NA | NA |
| 75 | Atrial Fibrillation | 7.74% | 3.39% | 12.79% | 10.73% | 4.97% | NA | NA | NA |
| 75 | Cancer | 14.74% | 0.00% | 100.00% | 6.61% | 100.00% | NA | NA | NA |
| 75 | Heart Failure | 4.98% | 2.08% | 9.83% | 12.04% | 2.57% | NA | NA | NA |
| 75 | Chronic Pain | 29.56% | 19.94% | 41.26% | 87.32% | 24.42% | NA | NA | NA |
| 75 | Pulmonary Disease | 6.84% | 6.87% | 100.00% | 78.04% | 0.00% | NA | NA | NA |
| 75 | Chronic Viral Hepatitis B | 0.02% | 0.03% | <3% | <3% | <3% | NA | NA | NA |
| 75 | Cirrhosis | 1.01% | 0.74% | 0.93% | 1.60% | 0.75% | NA | NA | NA |
| 75 | Dementia | 2.01% | 1.20% | 3.14% | 2.34% | 1.79% | NA | NA | NA |
| 75 | Depression | 24.85% | 28.75% | 32.79% | 44.63% | 27.69% | NA | NA | NA |
| 75 | Diabetes | 28.45% | 13.28% | 23.04% | 67.88% | 14.69% | NA | NA | NA |
| 75 | Epilepsy | 1.49% | 1.57% | 1.86% | 3.04% | 1.68% | NA | NA | NA |
| 75 | Hypertension | 100.00% | 0.00% | 62.20% | 68.99% | 0.00% | NA | NA | NA |
| 75 | Hypothyroidism | 9.63% | 9.79% | 11.08% | 14.05% | 10.12% | NA | NA | NA |
| 75 | IBD | 1.68% | 1.81% | 2.72% | 2.55% | 2.76% | NA | NA | NA |
| 75 | IBS | 2.19% | 2.05% | 3.14% | 5.25% | 2.28% | NA | NA | NA |
| 75 | Multiple Sclerosis | 0.24% | 0.26% | 0.16% | 0.25% | 0.21% | NA | NA | NA |
| 75 | MI | 11.28% | 4.45% | 14.52% | 18.48% | 5.05% | NA | NA | NA |
| 75 | Parkinson's Disease | 0.83% | 0.59% | 1.41% | 1.12% | 0.98% | NA | NA | NA |
| 75 | PUD | 2.64% | 1.80% | 4.79% | 5.29% | 2.20% | NA | NA | NA |
| 75 | PVD | 7.78% | 4.72% | 12.52% | 11.89% | 5.91% | NA | NA | NA |
| 75 | Psoriasis | 1.28% | 0.97% | 1.60% | 2.11% | 1.19% | NA | NA | NA |
| 75 | Rheumatoid Arthritis | 3.47% | 2.78% | 5.97% | 6.84% | 3.12% | NA | NA | NA |
| 75 | Schizophrenia | 0.73% | 1.53% | 0.75% | 1.60% | 1.06% | NA | NA | NA |
| 75 | Severe Constipation | 9.03% | 5.95% | 16.78% | 19.00% | 8.78% | NA | NA | NA |
| 75 | Stroke | 8.69% | 3.37% | 10.90% | 11.95% | 4.54% | NA | NA | NA |
| 90 | n | 136,801 | 16,909 | 2,149 | 54,820 | 4,119 | NA | NA | NA |
| 90 | Age | 51.09 | 59.51 | 63.42 | 60.07 | 61.47 | NA | NA | NA |
| 90 | GENDER = Female | 59.06% | 53.95% | 53.00% | 44.57% | 60.69% | NA | NA | NA |
| 90 | Alcohol Misuse | 4.54% | 9.89% | 14.47% | 4.68% | 9.57% | NA | NA | NA |
| 90 | Asthma | 11.71% | 24.68% | 22.94% | 11.97% | 25.01% | NA | NA | NA |
| 90 | Atrial Fibrillation | 1.21% | 4.28% | 8.24% | 3.59% | 4.73% | NA | NA | NA |
| 90 | Cancer | 7.03% | 0.00% | 14.43% | 10.77% | 100.00% | NA | NA | NA |
| 90 | Heart Failure | 0.80% | 5.10% | 5.72% | 2.34% | 4.78% | NA | NA | NA |
| 90 | Chronic Pain | 15.37% | 78.66% | 72.87% | 12.72% | 81.33% | NA | NA | NA |
| 90 | Pulmonary Disease | 6.08% | 16.88% | 22.94% | 8.62% | 21.10% | NA | NA | NA |
| 90 | Chronic Viral Hepatitis B | 0.04% | 0.07% | <3% | 0.04% | <3% | NA | NA | NA |
| 90 | Cirrhosis | 0.67% | 2.70% | 1.26% | 1.05% | 2.52% | NA | NA | NA |
| 90 | Dementia | 0.25% | 1.05% | 4.14% | 0.38% | 1.21% | NA | NA | NA |
| 90 | Depression | 34.88% | 79.06% | 73.20% | 16.72% | 82.57% | NA | NA | NA |
| 90 | Diabetes | 9.55% | 71.84% | 0.00% | 18.63% | 54.67% | NA | NA | NA |
| 90 | Epilepsy | 1.81% | 3.14% | 9.59% | 1.52% | 3.64% | NA | NA | NA |
| 90 | Hypertension | 0.00% | 89.52% | 80.78% | 100.00% | 54.33% | NA | NA | NA |
| 90 | Hypothyroidism | 7.26% | 10.79% | 10.10% | 6.59% | 11.97% | NA | NA | NA |
| 90 | IBD | 1.96% | 2.22% | 2.70% | 1.55% | 3.69% | NA | NA | NA |
| 90 | IBS | 2.15% | 5.26% | 4.70% | 1.72% | 5.56% | NA | NA | NA |
| 90 | Multiple Sclerosis | 0.38% | 0.59% | 0.79% | 0.24% | 0.70% | NA | NA | NA |
| 90 | MI | 2.00% | 12.31% | 11.17% | 6.99% | 9.10% | NA | NA | NA |
| 90 | Parkinson's Disease | 0.23% | 0.72% | 1.81% | 0.33% | 1.19% | NA | NA | NA |
| 90 | PUD | 1.34% | 3.65% | 4.84% | 1.82% | 4.22% | NA | NA | NA |
| 90 | PVD | 2.99% | 7.85% | 9.91% | 4.83% | 7.53% | NA | NA | NA |
| 90 | Psoriasis | 1.01% | 2.17% | 2.23% | 1.32% | 2.33% | NA | NA | NA |
| 90 | Rheumatoid Arthritis | 2.28% | 5.25% | 6.47% | 2.49% | 6.26% | NA | NA | NA |
| 90 | Schizophrenia | 1.45% | 2.32% | 1.63% | 0.57% | 2.14% | NA | NA | NA |
| 90 | Severe Constipation | 4.51% | 13.82% | 20.10% | 4.45% | 18.31% | NA | NA | NA |
| 90 | Stroke | 1.42% | 5.67% | 100.00% | 3.13% | 4.71% | NA | NA | NA |

Figure S6. Identification of prominent condition(s) by cluster and eGFR category.

The red marks indicate which conditions have a prevalence of ≥20% and an O/E ratio of ≥2.

A. SCREAM B. SAIL


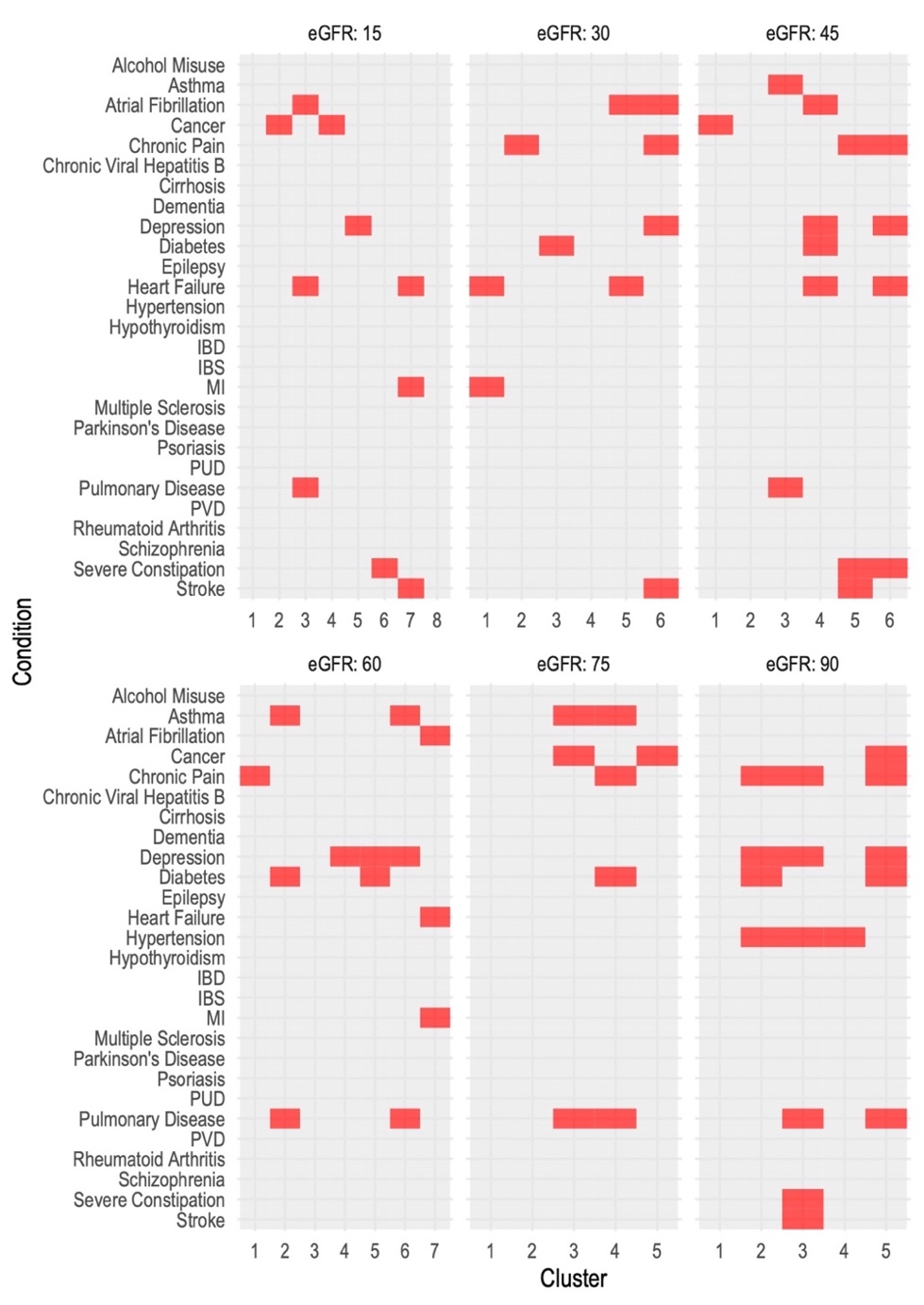

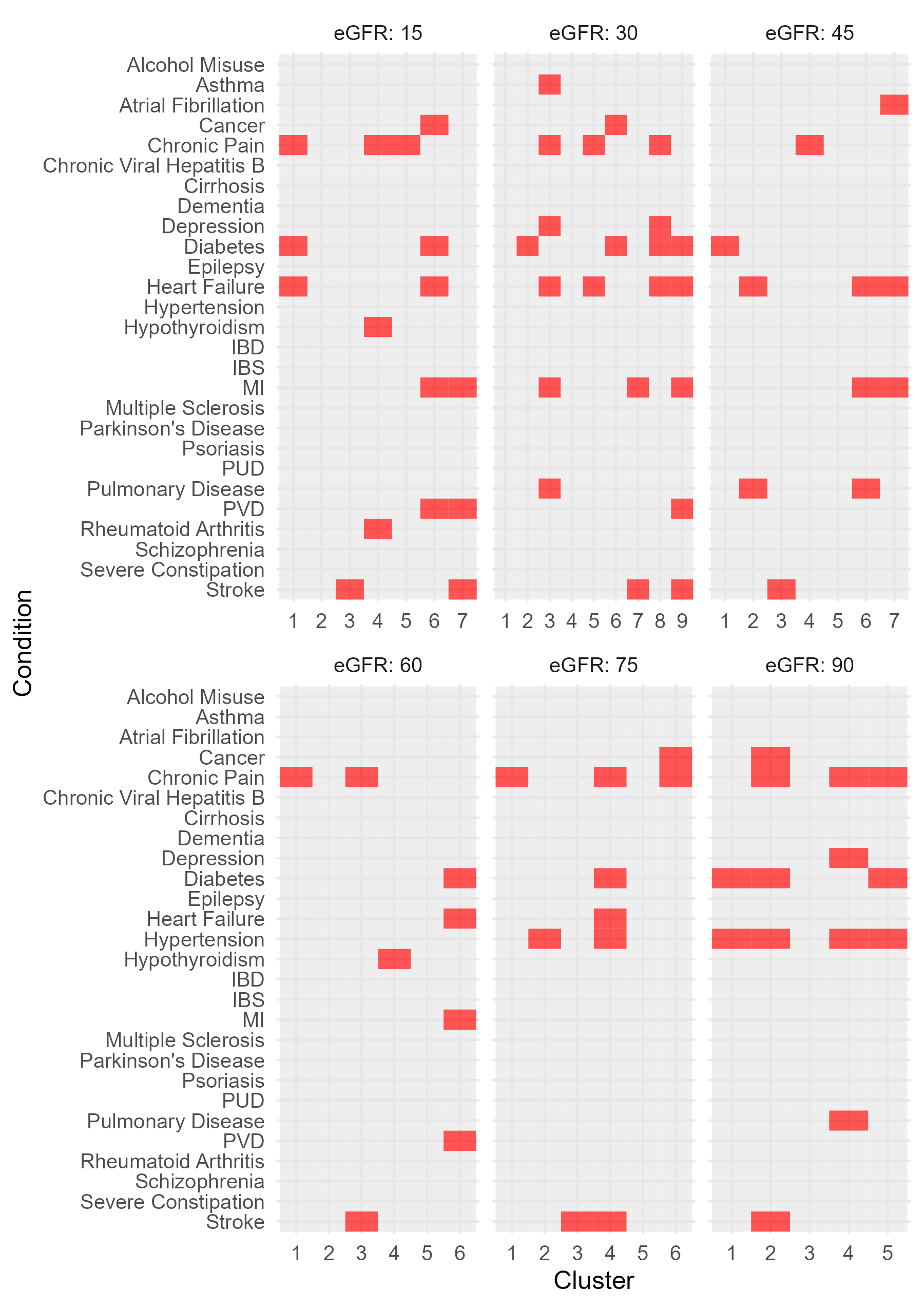


Figure S7A. Age-stratified prominent conditions in SCREAM (≥65 and <65 years).

Figure S7B. Age-stratified prominent conditions in SAIL (<65 and ≥65 years).

| **eGFR category** | **Median follow-up time**  **(years: interquartile interval)** |
| --- | --- |
| 15 | 1.94 (1.87-2.03) |
| 30 | 4.21 (4.11-4.32) |
| 45 | 5.10 (5.04-5.15) |
| 60 | 5.44 (5.40-5.48) |
| 75 | 5.70 (5.67-5.73) |
| 90 | 6.32 (6.30-6.34) |

Table S3A. Median follow-up time in SCREAM

| **eGFR category** | **Median follow-up time**  **(years: interquartile interval)** |
| --- | --- |
| 15 | 5.51 (5.32-5.68) |
| 30 | 6.68 (6.60-6.77) |
| 45 | 7.55 (7.50-7.60) |
| 60 | 8.22 (8.18-8.26) |
| 75 | 8.14 (8.11-8.17) |
| 90 | 7.45 (7.42-7.47) |

Table S3B. Median follow-up time in SAIL

Figure S8A. Adverse event rates by cluster in SCREAM. Error bars represent 95% confidence intervals

Figure S8B. Adverse event rates by cluster in SAIL. Error bars represent 95% confidence intervals

Figure S9A1. Standardised regression survival curves in eGFR 15 category in SCREAM

Figure S9A2. Standardised regression survival curves in eGFR 30 category in SCREAM

Figure S9A3. Standardised regression survival curves in eGFR 45 category in SCREAM

Figure S9A4. Standardised regression survival curves in eGFR 60 category in SCREAM

Figure S9A5. Standardised regression survival curves in eGFR 75 category in SCREAM

Figure S9A6. Standardised regression survival curves in eGFR 90 category in SCREAM

Figure S9B1. Standardised regression survival curves in eGFR 15 category in SAIL

Figure S9B2. Standardised regression survival curves in eGFR 30 category in SAIL

Figure S9B3. Standardised regression survival curves in eGFR 45 category in SAIL

Figure S9B4. Standardised regression survival curves in eGFR 60 category in SAIL

Figure S9B5. Standardised regression survival curves in eGFR 75 category in SAIL

Figure S9B6. Standardised regression survival curves in eGFR 90 category in SAIL

Figure S10A. Predictive performance of clusters for adverse outcomes in SCREAM using area under curve (AUC). ACM, all-cause mortality. MACE3, major adverse cardiovascular events (myocardial infarction, stroke, cardiovascular death), MACE4, (myocardial infarction, stroke, cardiovascular death, heart failure hospitalisation). Time is measured in years

Figure S10B. Predictive performance of clusters for adverse outcomes in SAIL using area under curve (AUC). ACM, all-cause mortality. MACE3, major adverse cardiovascular events (myocardial infarction, stroke, cardiovascular death), MACE4, (myocardial infarction, stroke, cardiovascular death, heart failure hospitalisation). Time is measured in years

Figure S11A. Predictive performance of clusters for adverse outcomes in SCREAM using Brier score. ACM, all-cause mortality. MACE3, major adverse cardiovascular events (myocardial infarction, stroke, cardiovascular death), MACE4, (myocardial infarction, stroke, cardiovascular death, heart failure hospitalisation). Time is measured in years

Figure S11B. Predictive performance of clusters for adverse outcomes in SAIL using Brier score. ACM, all-cause mortality. MACE3, major adverse cardiovascular events (myocardial infarction, stroke, cardiovascular death), MACE4, (myocardial infarction, stroke, cardiovascular death, heart failure hospitalisation). Time is measured in years

Figure S12A. Forest plot showing the risk of all-cause mortality (ACM) and MACE by cluster allocation in SCREAM. Hazard ratios are adjusted for sex and age


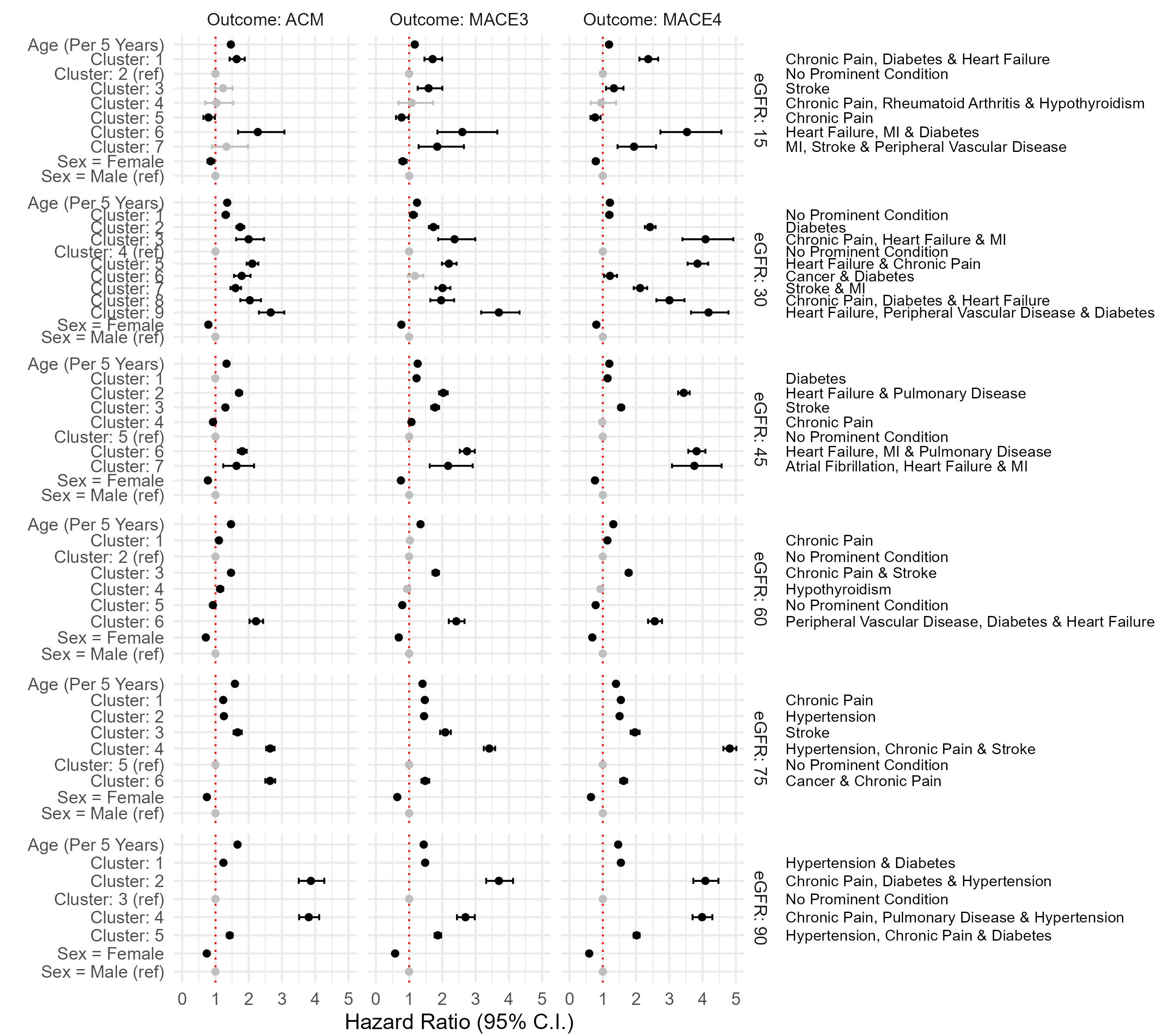


Figure S12B. Forest plot showing the risk of all-cause mortality (ACM) and MACE by cluster allocation in SAIL. Hazard ratios are adjusted for sex and age

Table S4A. Risk of adverse outcomes by cluster in SCREAM. Cox models adjusted for age and sex. ACM, all-cause mortality, MACE3, major adverse cardiovascular events (myocardial infarction, stroke, cardiovascular death), MACE4, (myocardial infarction, stroke, cardiovascular death, heart failure hospitalisation)

| **eGFR 15** | **ACM** | **MACE3** | **MACE4** |
| --- | --- | --- | --- |
| Age (Per 5 Years) | 1.462 (1.418-1.509) | 1.170 (1.138-1.202) | 1.188 (1.162-1.214) |
| Cluster: 1 | 1.638 (1.425-1.881) | 1.706 (1.460-1.993) | 2.366 (2.101-2.664) |
| Cluster: 2 (ref) | --- | --- | --- |
| Cluster: 3 | 1.225 (0.991-1.515) | 1.585 (1.257-1.998) | 1.334 (1.097-1.622) |
| Cluster: 4 | 1.031 (0.691-1.538) | 1.080 (0.678-1.719) | 0.948 (0.642-1.399) |
| Cluster: 5 | 0.791 (0.637-0.984) | 0.773 (0.605-0.988) | 0.767 (0.630-0.935) |
| Cluster: 6 | 2.272 (1.680-3.074) | 2.599 (1.850-3.651) | 3.532 (2.733-4.565) |
| Cluster: 7 | 1.332 (0.897-1.978) | 1.845 (1.286-2.648) | 1.937 (1.443-2.601) |
| Sex = Female | 0.855 (0.758-0.965) | 0.808 (0.706-0.924) | 0.788 (0.709-0.875) |
| Sex = Male (ref) | --- | --- | --- |
| **eGFR 30** |  |  |  |
| Age (Per 5 Years) | 1.353 (1.335-1.372) | 1.238 (1.219-1.257) | 1.213 (1.199-1.228) |
| Cluster: 1 | 1.307 (1.215-1.406) | 1.125 (1.026-1.233) | 1.195 (1.107-1.291) |
| Cluster: 2 | 1.743 (1.628-1.866) | 1.731 (1.594-1.878) | 2.420 (2.263-2.588) |
| Cluster: 3 | 1.998 (1.621-2.462) | 2.366 (1.873-2.990) | 4.088 (3.393-4.926) |
| Cluster: 4 (ref) | --- | --- | --- |
| Cluster: 5 | 2.106 (1.938-2.288) | 2.196 (1.986-2.427) | 3.847 (3.549-4.170) |
| Cluster: 6 | 1.789 (1.558-2.054) | 1.172 (0.966-1.422) | 1.216 (1.036-1.427) |
| Cluster: 7 | 1.602 (1.452-1.769) | 2.004 (1.792-2.241) | 2.122 (1.929-2.334) |
| Cluster: 8 | 2.034 (1.747-2.367) | 1.963 (1.637-2.353) | 3.005 (2.611-3.458) |
| Cluster: 9 | 2.661 (2.307-3.070) | 3.698 (3.166-4.319) | 4.179 (3.653-4.779) |
| Sex = Female | 0.787 (0.750-0.825) | 0.765 (0.723-0.811) | 0.805 (0.768-0.843) |
| Sex = Male (ref) | --- | --- | --- |
| **eGFR 45** |  |  |  |
| Age (Per 5 Years) | 1.333 (1.320-1.346) | 1.256 (1.243-1.270) | 1.196 (1.186-1.206) |
| Cluster: 1 | 0.994 (0.950-1.039) | 1.223 (1.156-1.293) | 1.145 (1.093-1.199) |
| Cluster: 2 | 1.708 (1.622-1.799) | 2.026 (1.900-2.160) | 3.434 (3.264-3.613) |
| Cluster: 3 | 1.298 (1.228-1.373) | 1.779 (1.663-1.902) | 1.550 (1.464-1.641) |
| Cluster: 4 | 0.926 (0.878-0.976) | 1.071 (1.002-1.143) | 0.981 (0.929-1.036) |
| Cluster: 5 (ref) | --- | --- | --- |
| Cluster: 6 | 1.803 (1.676-1.940) | 2.740 (2.523-2.976) | 3.820 (3.571-4.086) |
| Cluster: 7 | 1.633 (1.235-2.161) | 2.172 (1.620-2.912) | 3.753 (3.081-4.572) |
| Sex = Female | 0.772 (0.748-0.796) | 0.753 (0.726-0.781) | 0.764 (0.742-0.787) |
| Sex = Male (ref) | --- | --- | --- |
| **eGFR: 60** |  |  |  |
| Age (Per 5 Years) | 1.467 (1.455-1.479) | 1.342 (1.330-1.354) | 1.315 (1.306-1.324) |
| Cluster: 1 | 1.105 (1.070-1.142) | 1.025 (0.989-1.064) | 1.139 (1.106-1.173) |
| Cluster: 2 (ref) | --- | --- | --- |
| Cluster: 3 | 1.468 (1.394-1.545) | 1.797 (1.702-1.897) | 1.777 (1.698-1.860) |
| Cluster: 4 | 1.140 (1.054-1.234) | 0.939 (0.854-1.034) | 0.928 (0.857-1.005) |
| Cluster: 5 | 0.923 (0.893-0.953) | 0.794 (0.764-0.824) | 0.785 (0.761-0.810) |
| Cluster: 6 | 2.218 (2.023-2.433) | 2.418 (2.193-2.666) | 2.562 (2.361-2.781) |
| Sex = Female | 0.710 (0.692-0.728) | 0.688 (0.668-0.708) | 0.685 (0.669-0.702) |
| Sex = Male (ref) | --- | --- | --- |
| **eGFR: 75** |  |  |  |
| Age (Per 5 Years) | 1.586 (1.575-1.597) | 1.403 (1.393-1.414) | 1.397 (1.388-1.405) |
| Cluster: 1 | 1.234 (1.192-1.276) | 1.471 (1.420-1.524) | 1.542 (1.497-1.589) |
| Cluster: 2 | 1.254 (1.213-1.295) | 1.449 (1.400-1.499) | 1.505 (1.462-1.549) |
| Cluster: 3 | 1.663 (1.545-1.790) | 2.087 (1.932-2.254) | 1.967 (1.838-2.106) |
| Cluster: 4 | 2.646 (2.523-2.776) | 3.410 (3.244-3.586) | 4.816 (4.625-5.015) |
| Cluster: 5 (ref) | --- | --- | --- |
| Cluster: 6 | 2.641 (2.497-2.794) | 1.481 (1.368-1.603) | 1.624 (1.522-1.733) |
| Sex = Female | 0.742 (0.723-0.761) | 0.643 (0.626-0.660) | 0.644 (0.630-0.659) |
| Sex = Male (ref) | --- | --- | --- |
| **eGFR 90** |  |  |  |
| Age (Per 5 Years) | 1.663 (1.647-1.680) | 1.438 (1.425-1.451) | 1.463 (1.451-1.474) |
| Cluster: 1 | 1.239 (1.189-1.291) | 1.482 (1.425-1.541) | 1.543 (1.492-1.596) |
| Cluster: 2 | 3.870 (3.505-4.272) | 3.699 (3.317-4.125) | 4.083 (3.722-4.478) |
| Cluster: 3 (ref) | --- | --- | --- |
| Cluster: 4 | 3.805 (3.516-4.119) | 2.694 (2.439-2.974) | 3.985 (3.699-4.293) |
| Cluster: 5 | 1.429 (1.350-1.512) | 1.862 (1.768-1.962) | 2.016 (1.929-2.106) |
| Sex = Female | 0.739 (0.716-0.764) | 0.579 (0.560-0.598) | 0.591 (0.575-0.608) |
| Sex = Male (ref) | --- | --- | --- |

Table S4B. Risk of adverse outcomes by cluster in SAIL. Cox models adjusted for age and sex. ACM, all-cause mortality, MACE3, major adverse cardiovascular events (myocardial infarction, stroke, cardiovascular death), MACE4, (myocardial infarction, stroke, cardiovascular death, heart failure hospitalisation)

| **eGFR 15** | **ACM** | **MACE3** | **MACE4** |
| --- | --- | --- | --- |
| Age (Per 5 Years) | 1.072 (1.045-1.100) | 1.041 (1.017-1.066) | 1.047 (1.029-1.066) |
| Cluster: 1 (ref) | — | — | — |
| Cluster: 2 | 1.145 (0.899-1.458) | 1.113 (0.884-1.402) | 1.148 (0.968-1.362) |
| Cluster: 3 | 1.665 (1.297-2.137) | 1.526 (1.196-1.947) | 2.796 (2.375-3.293) |
| Cluster: 4 | 1.023 (0.789-1.326) | 1.049 (0.820-1.341) | 0.850 (0.699-1.034) |
| Cluster: 5 | 1.320 (1.065-1.635) | 1.219 (0.993-1.496) | 1.241 (1.067-1.445) |
| Cluster: 6 | 1.770 (1.382-2.267) | 1.680 (1.325-2.130) | 1.374 (1.135-1.662) |
| Cluster: 7 | 2.884 (2.213-3.757) | 2.962 (2.307-3.805) | 3.103 (2.546-3.781) |
| Cluster: 8 | 1.430 (1.162-1.761) | 1.337 (1.093-1.634) | 1.119 (0.955-1.312) |
| Sex = Female | 0.804 (0.704-0.919) | 0.810 (0.713-0.920) | 0.874 (0.794-0.961) |
| Sex = Male (ref) | — | — | — |
| **eGFR 30** |  |  |  |
| Age (Per 5 Years) | 1.075 (1.061-1.089) | 1.045 (1.033-1.057) | 1.055 (1.045-1.064) |
| Cluster: 1 | 1.898 (1.747-2.062) | 1.809 (1.671-1.958) | 2.448 (2.308-2.597) |
| Cluster: 2 | 1.061 (0.976-1.154) | 1.062 (0.982-1.150) | 1.029 (0.968-1.093) |
| Cluster: 3 | 1.361 (1.257-1.474) | 1.297 (1.203-1.399) | 1.268 (1.197-1.343) |
| Cluster: 4 (ref) | — | — | — |
| Cluster: 5 | 2.230 (2.041-2.436) | 1.997 (1.833-2.175) | 3.425 (3.220-3.642) |
| Cluster: 6 | 1.378 (1.182-1.606) | 1.348 (1.165-1.560) | 1.583 (1.423-1.761) |
| Sex = Female | 0.846 (0.801-0.893) | 0.852 (0.809-0.897) | 0.841 (0.810-0.874) |
| Sex = Male (ref) | — | — | — |
| **eGFR 45** |  |  |  |
| Age (Per 5 Years) | 1.123 (1.112-1.134) | 1.070 (1.061-1.079) | 1.078 (1.071-1.085) |
| Cluster: 1 | 0.978 (0.927-1.032) | 0.950 (0.904-0.998) | 1.051 (1.013-1.091) |
| Cluster: 2 (ref) | — | — | — |
| Cluster: 3 | 1.302 (1.230-1.377) | 1.226 (1.164-1.292) | 1.512 (1.454-1.572) |
| Cluster: 4 | 1.995 (1.885-2.112) | 1.727 (1.637-1.822) | 2.815 (2.709-2.924) |
| Cluster: 5 | 1.202 (1.111-1.300) | 1.191 (1.107-1.281) | 1.154 (1.089-1.223) |
| Cluster: 6 | 1.290 (1.179-1.412) | 1.187 (1.090-1.292) | 1.844 (1.739-1.956) |
| Sex = Female | 0.767 (0.741-0.795) | 0.784 (0.759-0.810) | 0.760 (0.741-0.779) |
| Sex = Male (ref) | — | — | — |
| **eGFR 60** |  |  |  |
| Age (Per 5 Years) | 1.232 (1.221-1.243) | 1.132 (1.123-1.140) | 1.140 (1.134-1.147) |
| Cluster: 1 | 1.303 (1.254-1.354) | 1.226 (1.186-1.267) | 1.364 (1.330-1.400) |
| Cluster: 2 | 1.571 (1.494-1.653) | 1.400 (1.339-1.463) | 1.863 (1.803-1.924) |
| Cluster: 3 (ref) | — | — | — |
| Cluster: 4 | 0.969 (0.894-1.050) | 0.921 (0.861-0.986) | 1.020 (0.970-1.074) |
| Cluster: 5 | 1.691 (1.584-1.806) | 1.534 (1.451-1.622) | 1.715 (1.643-1.790) |
| Cluster: 6 | 1.320 (1.165-1.496) | 1.272 (1.145-1.413) | 1.686 (1.564-1.817) |
| Cluster: 7 | 2.230 (2.056-2.419) | 1.940 (1.801-2.090) | 2.889 (2.738-3.048) |
| Sex = Female | 0.714 (0.692-0.737) | 0.724 (0.705-0.744) | 0.716 (0.701-0.731) |
| Sex = Male (ref) | — | — | — |
| **eGFR 75** |  |  |  |
| Age (Per 5 Years) | 1.388 (1.375-1.400) | 1.234 (1.226-1.242) | 1.247 (1.240-1.254) |
| Cluster: 1 (ref) | — | — | — |
| Cluster: 2 | 0.684 (0.656-0.712) | 0.732 (0.709-0.755) | 0.691 (0.674-0.708) |
| Cluster: 3 | 1.137 (1.024-1.262) | 0.995 (0.909-1.090) | 1.194 (1.117-1.277) |
| Cluster: 4 | 1.792 (1.686-1.904) | 1.542 (1.463-1.625) | 1.897 (1.824-1.972) |
| Cluster: 5 | 0.612 (0.559-0.670) | 0.647 (0.602-0.694) | 0.641 (0.606-0.677) |
| Sex = Female | 0.676 (0.653-0.700) | 0.653 (0.636-0.672) | 0.657 (0.643-0.671) |
| Sex = Male (ref) | — | — | — |
| **eGFR 90** |  |  |  |
| Age (Per 5 Years) | 1.462 (1.441-1.483) | 1.284 (1.272-1.297) | 1.320 (1.310-1.330) |
| Cluster: 1 (ref) | — | — | — |
| Cluster: 2 | 2.793 (2.594-3.007) | 2.331 (2.209-2.460) | 2.612 (2.503-2.726) |
| Cluster: 3 | 2.787 (2.377-3.267) | 2.485 (2.202-2.804) | 2.414 (2.184-2.668) |
| Cluster: 4 | 1.366 (1.284-1.454) | 1.327 (1.271-1.385) | 1.393 (1.346-1.442) |
| Cluster: 5 | 1.908 (1.636-2.225) | 1.529 (1.357-1.722) | 1.918 (1.756-2.095) |
| Sex = Female | 0.564 (0.534-0.596) | 0.551 (0.530-0.573) | 0.581 (0.564-0.599) |
| Sex = Male (ref) | — | — | — |
